# Automatic feature engineering for catalyst design using small data without prior knowledge of target catalysis

**DOI:** 10.1038/s42004-023-01086-y

**Published:** 2024-01-12

**Authors:** Toshiaki Taniike, Aya Fujiwara, Sunao Nakanowatari, Fernando García-Escobar, Keisuke Takahashi

**Affiliations:** 1 Graduate School of Advanced Science and Technology, Japan Advanced Institute of Science and Technology, 1-1 Asahidai, Nomi, Ishikawa, 923-1292 Japan; 2 Department of Chemistry, Hokkaido University, North 10, West 8, Sapporo, 060-0810 Japan

**Keywords:** Heterogeneous catalysis, Cheminformatics, Computational methods, Structure prediction

## Abstract

The empirical aspect of descriptor design in catalyst informatics, particularly when confronted with limited data, necessitates adequate prior knowledge for delving into unknown territories, thus presenting a logical contradiction. This study introduces a technique for automatic feature engineering (AFE) that works on small catalyst datasets, without reliance on specific assumptions or pre-existing knowledge about the target catalysis when designing descriptors and building machine-learning models. This technique generates numerous features through mathematical operations on general physicochemical features of catalytic components and extracts relevant features for the desired catalysis, essentially screening numerous hypotheses on a machine. AFE yields reasonable regression results for three types of heterogeneous catalysis: oxidative coupling of methane (OCM), conversion of ethanol to butadiene, and three-way catalysis, where only the training set is swapped. Moreover, through the application of active learning that combines AFE and high-throughput experimentation for OCM, we successfully visualize the machine’s process of acquiring precise recognition of the catalyst design. Thus, AFE is a versatile technique for data-driven catalysis research and a key step towards fully automated catalyst discoveries.

## Abstract (web summary)

Descriptor design in catalyst informatics necessitates adequate prior knowledge for delving into unknown territories, particularly when confronted with limited data, thus presenting a logical contradiction. Here, the authors report a technique for automatic feature engineering that works on small catalyst datasets without reliance on pre-existing knowledge about the target catalysis.

## Introduction

Over the years, the trajectory of natural science has been conventionally steered by the intuition of individual researchers, guiding the formulation of hypotheses and their subsequent validation through experimentation. However, with the advent of a data-driven approach, this paradigm is now shifting, challenging established norms and registering significant success across diverse fields, including catalysis^1–4^. Within the realm of data-driven catalysis research, particularly in the context of experimental catalyst discoveries, the limited availability of data characterized by both sufficient quantity and quality for effective machine learning (ML) presents a major hurdle^5–8^. In this context, data typically assume the form of tabular datasets comprising observations (e.g., catalyst samples) and parameters describing these observations (properties of catalysts), commonly referred to as features or descriptors when employed to predict a specific target variable (performance of catalysts) within the framework of supervised ML. In the field of catalysis, data are predominantly categorized into small data, seldom surpassing a thousand observations. This characteristic renders the data unsuitable for the deployment of elaborate ML models with a multitude of adjustable parameters necessary to capture intricate trends. Thus, the design of descriptors that encapsulate the essence of catalysis is imperative for the efficient and accurate capturing of data trends using simple ML models. However, except in limited cases of crystal structures^9^ and organic reactions^10^, the data limitation has rendered the application of deep learning impractical, prompting researchers to address the fundamental issue of descriptor design in ML^1,11^. Indeed, descriptor design based on individual researchers’ insights into structure–activity relationships, such as the *d*-band center in metal nanoalloys^12^ and the buried volume in organometallic asymmetric catalysis^13^, constitutes a key aspect of the progress in catalyst informatics^6,14–16^. However, such descriptor design is generally challenging and performed ad hoc, as it requires profound domain knowledge to identify all pertinent factors for the target catalysis^1,16,17^. In particular, practical solid catalysts constitute multiple components that are structured in an ill-defined manner, and the complex interplay of these components over multiple spatiotemporal scales results in the overall catalytic performance^18,19^. This intricacy, coupled with data scarcity, elevates the difficulty of crafting descriptors in catalysis, when compared to other fields.

To surmount these challenges, in this study, we developed an automatic feature engineering (AFE) technique that works on small data for complex materials, such as solid catalysts, without requiring any prior knowledge of the target system. The AFE is a structured pipeline of (i) assigning a series of features to materials of arbitrary compositions, (ii) synthesizing numerous higher-order features considering nonlinear and combinatorial effects, and (iii) selecting a feature subset in the context of supervised ML. This study explores the applicability of AFE across various heterogeneous catalysis scenarios, each characterized by distinct catalyst designs. Furthermore, an extension of AFE to active learning, coupled with high-throughput experimentation (HTE), is implemented to comprehend catalyst design rules and streamline catalyst discoveries.

## Results and discussion

### Automatic feature engineering

**Fig. 1:**
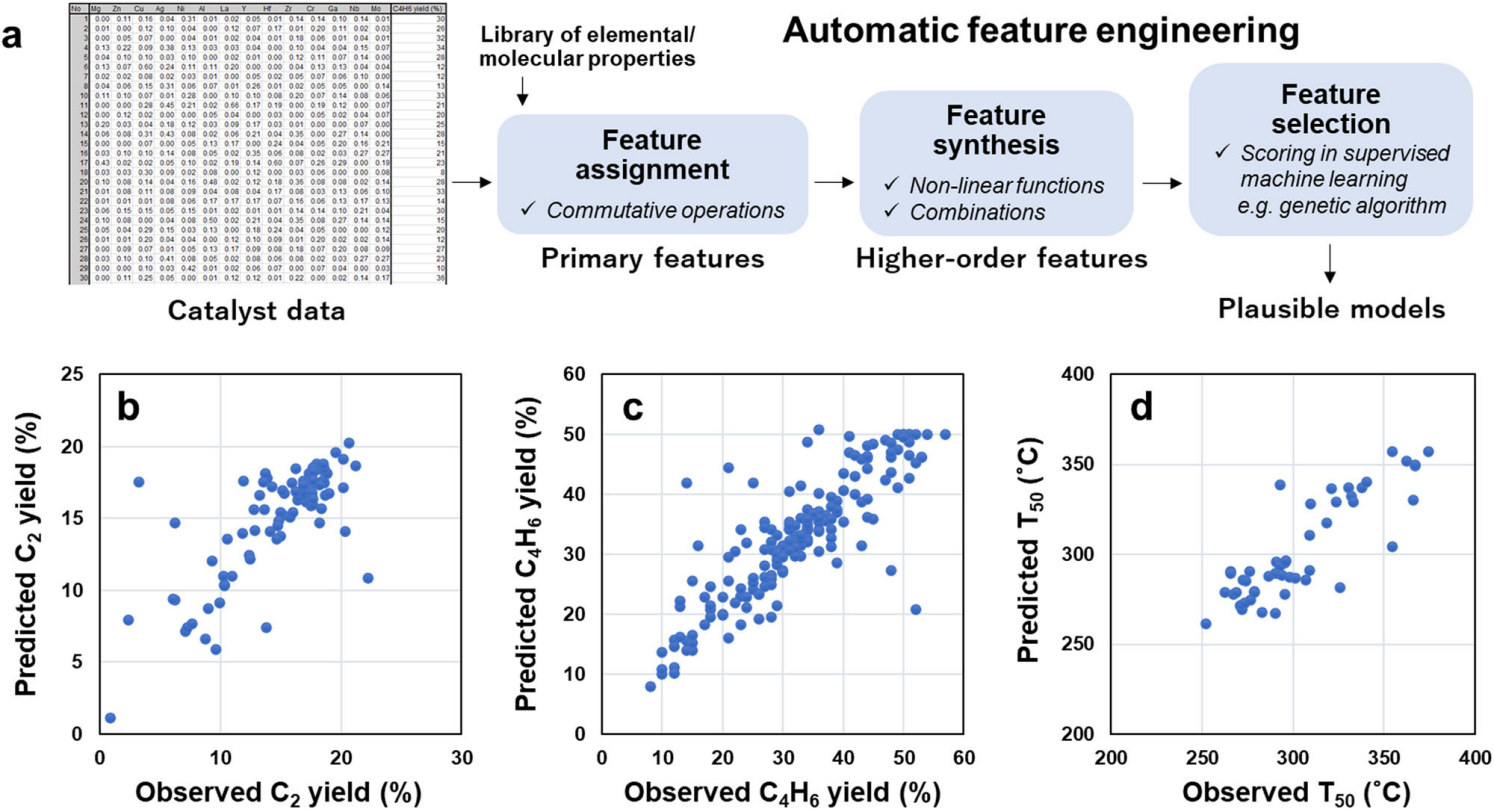
Automatic feature engineering (AFE) and its demonstration. **a** Schematic of the AFE pipeline. Prediction of (**b**) C_2_ yields in the oxidative coupling of methane (OCM), (**c**) butadiene yields in ethanol conversion, and (**d**) light-off temperatures for NO conversion in three-way catalysis. Eight features that minimized the mean absolute error (MAE) in leave-one-out cross-validation (LOOCV) with Huber regression were selected from 5568 first-order features.

Figure 1a illustrates the workflow of AFE. Here, we consider supported multi-element catalysts as typical examples, wherein the dataset comprises elemental composition and performance data for individual catalysts. While the straightforward and commonly employed approach involves directly using elemental compositions as descriptors in constructing an ML model, this neglects the physical properties of elements, leading to drawbacks such as insufficient prediction accuracy and an inability to handle elements absent in the training data. However, crafting physically meaningful features of catalysts remains challenging, as proposing these features is equivalent to hypothesizing their relevance in the target catalysis. The proposed AFE technique is based on the premise of our scarce knowledge of a system, a common characteristic in today’s research and development landscape with continually emerging demands over a short period. The first step in AFE involves assigning primary features to catalysts by computing commutative operations of a feature library, such as a maximum and weighted average. This accounts for notational order invariance (e.g., features of Li‒W must be equal to those of W‒Li) and the elemental compositions of catalysts (e.g., the features of Li‒Li‒W must be differentiated from those of Li‒W‒W)^20^. The feature library collects all possible features of the catalyst constituents (such as the properties of elements and molecules) from all available sources, assuming that all features are equally probable. In the next step, higher-order features, also called compound features^21–23^, are synthesized. These features are arbitrary functions of primary features (first order) and products of two or more of these functions (second or higher order), addressing the nonlinear and combinatorial aspects of the problem. This compensates for the limited expressive power of simple ML models suitable for small data. A detailed classification of different feature types is presented in Table S1. In the final step, the optimum feature combination that maximizes the performance of supervised ML is selected from a large pool of features (typically 10^3^‒10^6^). Hence, AFE generates a vast number of features (hypotheses) and recommends the most plausible combination within the context of supervised ML. While previous studies have employed preselected physical properties of elements to describe multi-element catalysts^24–26^, these properties have been hardly utilized to systematize feature engineering through the synthesis and screening of a large number of features. Herein, AFE was demonstrated using three HTE datasets of supported multi-element catalysts for different catalysis^27–32^ (Fig. 1b‒d; the datasets are given in Tables S2‒4). In particular, 5568 first-order features were constructed by applying eight types of commutative operations and 12 types of functions to 58 features of elements stored in XenonPy^33^. Then, eight features were selected to minimize the mean absolute error (MAE) in leave-one-out cross-validation (LOOCV) using Huber regression. Note that Huber regression is a linear regression method that employs the Huber loss instead of ordinary least squares to enhance robustness against outliers^34^. This approach not only mitigates the risk of overfitting on small data owing to its simplicity but also provides resilience against experimental errors and singular catalysts. Note that many of the generated features are inherently ineffective in describing the desired catalysis. However, given the limited knowledge and the fact that algorithm-based filtrations necessarily deteriorate the regression scores, filtering these features prior to feature selection is discouraged. Further details on this aspect are presented in the Methods section. In all cases, reasonable regression results evidenced the versatility of the method in tailoring the features for individual catalysis without prior knowledge (Fig. 1b‒d). The MAE values of the obtained models during training and CV were 1.69% and 1.73% in C_2_ yields, 3.77% and 3.93% in butadiene yields, and 11.2 °C and 11.9 °C in T_50_ of NO conversion, respectively. Notably, these MAE values are significantly smaller than the span of each target variable and comparable to the respective experimental errors. The remarkable accuracy of the AFE-generated models in CV was unattainable when using catalyst elemental compositions as descriptors, regardless of the ML methods and hyperparameter sets (Fig. S1). In particular, relatively complex methods such as support vector regression and random forest regression exhibited a tendency of overfitting with MAE in training significantly lower than that in CV. By contrast, AFE led to consistently low MAE values in both training and CV, offering a minimal set of engineered features suitable for capturing complex trends with limited data.

### Integration with active learning

**Fig. 2:**
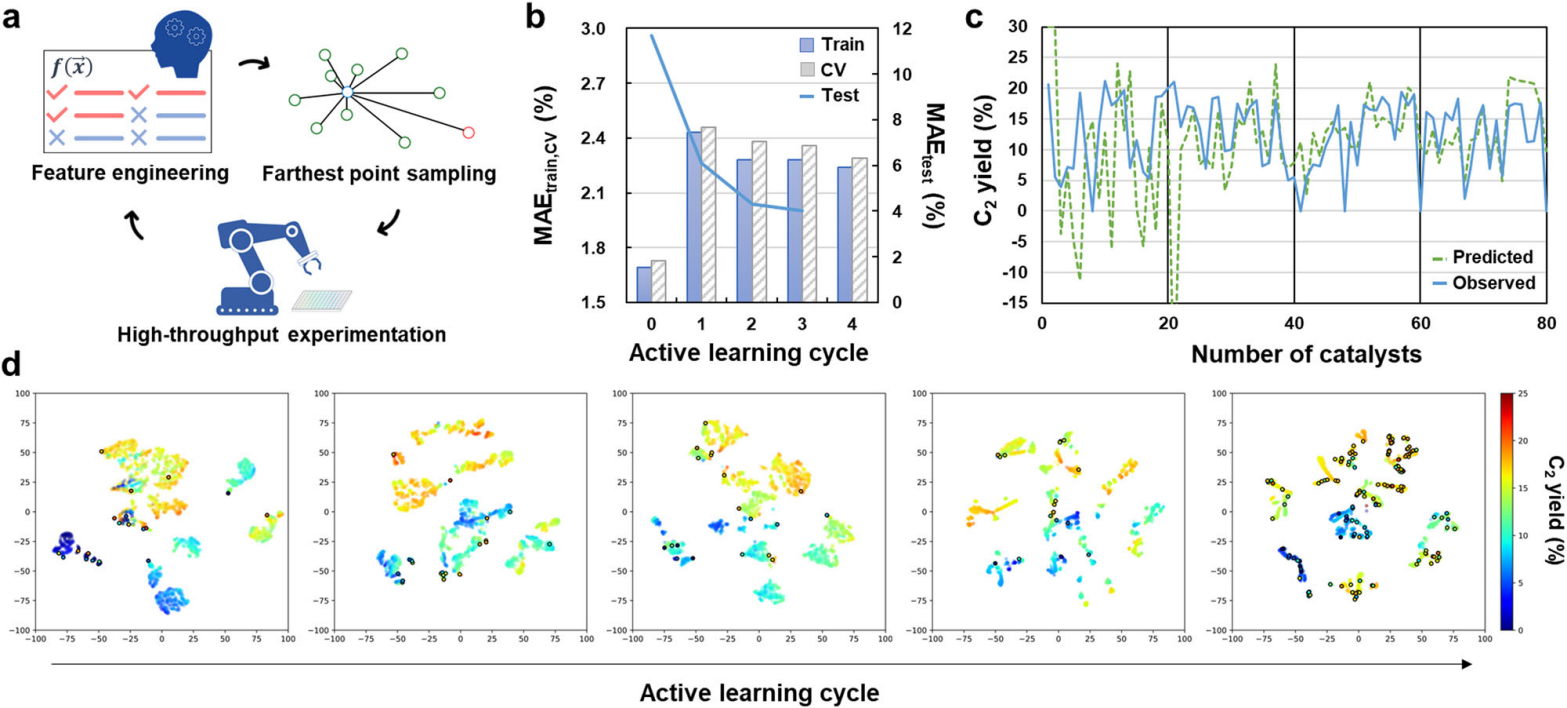
Active learning implemented for the OCM catalyst design. **a** Schematic of the active learning loop. The feature engineering was repeated five times with the data of 20 catalysts added per update. The model scores and the testing results are shown in (**b**) and (**c**), respectively. The deviation between predicted and observed C_2_ yields decreased monotonically throughout the active learning cycle. (**d**) Eight features were selected from 5568 first-order features to minimize the MAE in LOOCV with Huber regression. The development of the feature engineering and prediction is visualized based on t-distributed stochastic neighbor embedding (t-SNE). The circled data points are the test results except for the last cycle, which used the training data instead. The color reflects the predicted or observed C_2_ yield. Each t-SNE image delineates how the machine perceives the composition and performance of individual catalysts in each active learning cycle. The increase in the number of clusters during active learning signifies the evolution of the machine’s ability to discern diverse catalysts based on their distinct composition-performance relationships.

In scenarios where the available data are limited, researchers cannot disregard alternative hypotheses. Similarly, when the training data are either limited in size or constrained in the diversity of elemental compositions in catalysts, AFE proposes multiple models exhibiting similar scores, even though different feature sets are selected. Although these models demonstrate similar performance in explaining the training data, their predictive behaviors for unknown catalysts can vary significantly. In other words, many of these models are only locally fit, lacking the global characteristics necessary to explain the entire composition. An active learning strategy enables AFE to exclude locally fit models and identify a globally fit model, i.e., the true hypothesis set. Here, this was practised using the oxidative coupling of methane (OCM) dataset (Table S2). The dataset includes the C_2_ yield of catalysts with up to three elements selected from an element library and supported on BaO, each at a fixed amount^27^. Initially, eight first-order features were selected based on LOOCV-MAE in Huber regression on a given training dataset. Subsequently, 20 catalysts were prepared and evaluated through HTE, among which 18 catalysts were selected via farthest point sampling (FPS) in the selected feature space, and two were chosen based on their highest absolute errors in the regression. Note that FPS adds catalysts that are least similar to those in the training data within the selected feature space, which aids in efficiently excluding models lacking global characteristics. The obtained data were fed back to AFE to update the feature space (Fig. 2a). This process was repeated over four iterations, resulting in the addition of 80 new catalysts (Table S5). A more detailed procedure is presented in Figure S2. Figure 2b, c provides a summary of the relevant scores and individual test results, respectively. In the first cycle, the largest diversification of catalyst composition driven by FPS moderately increased the MAE_train,CV_ values, but subsequent cycles did not largely change these values. The final MAE_trainv,CV_ values (2.2‒2.3%) were higher than the typical experimental error (1.0‒2.0%), partly because the linear model failed to capture various 0% C_2_ yield data (any observed inactivity may be attributed to several reasons). Excluding these data points reduced the MAE_CV_ to ~1.9%. The changes in the test score were larger than those in the training and CV scores. Several extrapolations occurred during the first cycle, where the predicted yield was >30% or <0%, resulting in an extremely large MAE_test_. These extrapolations correspond to the model attempting to explain catalysts entirely beyond its original consideration. As the cycle progressed and the catalysts in the training dataset diversified sufficiently, these extrapolations disappeared, and the difference between the observations and predictions decreased monotonically. Pearson’s correlation coefficient between the regression models increased from 0.6 in Cycles 0 and 1 to 0.9 in Cycles 3 and 4, indicating the convergence of feature engineering toward a global model.

### Decoding machine’s perception

**Fig. 3:**
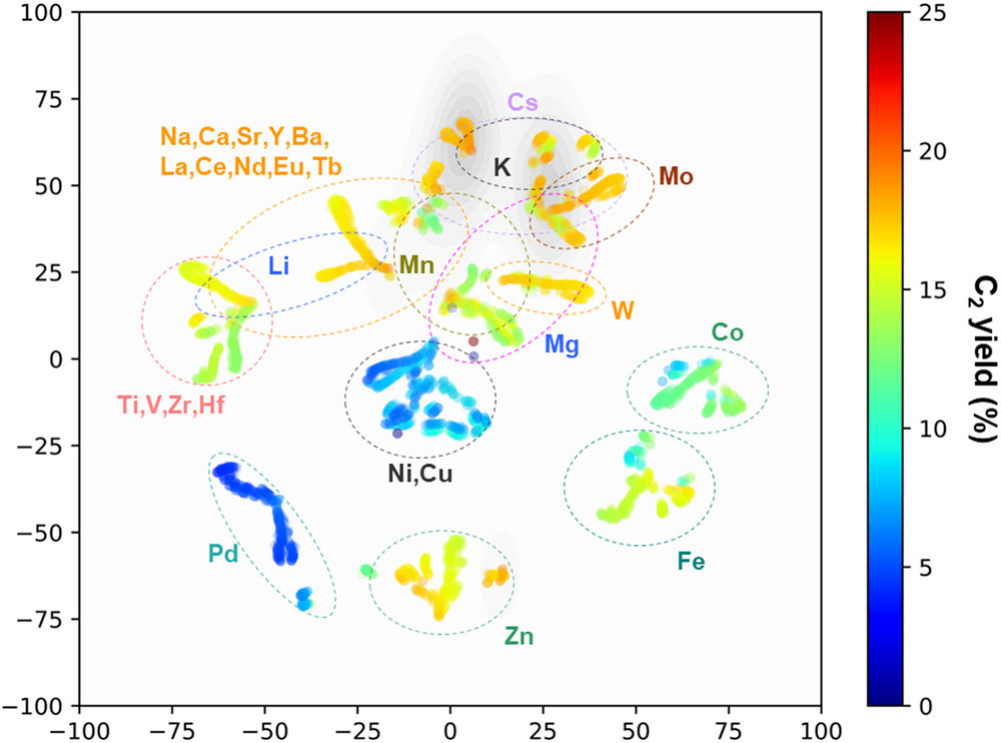
Machine perception of the OCM catalyst design. The feature space of the latest model is visualized by t-SNE, along with the Gaussian kernel density estimation for the C_2_ yield above 18%. The dotted lines indicate the regions where catalysts containing individual elements are concentrated. This visualization illustrates the machine’s perception in identifying the composition and performance of catalysts based on specific elements. It showcases common elements found in high and low-performing catalysts, similarities among elements within the feature space, and other pertinent insights.

Figure 2d visualizes the progress of feature engineering using t-distributed stochastic neighbor embedding (t-SNE)^35^, where the eight features selected during each active learning cycle were reduced in two dimensions, maintaining the pairwise similarities of the catalysts. This approach allowed us to monitor the evolution of the machine’s ability to perceive individual catalysts. The plot shows all 4060 catalysts in the library (including both tested and untested ones), with the color indicating the predicted C_2_ yield and circled data points representing the test results. Leveraging the advancements in active learning, the data were divided into a larger number of clusters, representing the machine’s process of refining a feature space to distinguish the catalysts better through distinct composition–performance relationships. Then, the question is how does the machine perceive the composition-performance relationships? This was addressed in two steps. First, the dataset was subjected to manual statistical analysis, as shown in Fig. S3. Early transition metals such as Mo and Zr and heavy alkali metals such as K and Cs are attributed high performance (Fig. S3a, b). This is because early transition metals can form oxometalate anions active for OCM when they are combined with Ba in the support or other supported elements with low electron affinity^28,36,37^. Alkali metals can enhance the C_2_ selectivity by strengthening the basicity of alkali earth metal oxides^38–40^. By contrast, late transition metals (excluding Zn with completely filled 3d orbitals) tend to decrease the C_2_ yield with increasing group number (Fig. S3a, c), as they act as combustion catalysts^41^. Next, keeping the abovementioned researcher’s observations in mind, the machine’s perception was interpreted by analyzing the distribution of individual elements in the feature space (Fig. S4). Figure 3 summarizes the regions where individual elements are concentrated after active learning, which decodes the machine perception. Late transition metals form separate clusters, whereas Mo and W are concentrated in narrow regions, indicating that the machine recognizes these elements as having differently significant impacts on the performance. By contrast, elements with a wide spatial distribution either have limited data points (e.g., La) or exhibit significantly different performance depending on their combination (e.g., Mg and Mn). Elements with overlapping distributions are not only similar in their physicochemical properties but also in their impact on the catalytic performance. For example, high-performing K and Cs have overlapping distributions, whereas the less-effective Li and Na are separated. These observations align with the researchers’ understanding acquired from Fig. S3. An application of the same analysis to the unselected feature set and the feature set selected before active learning (Fig. S5) revealed the essentiality of both feature engineering and active learning in achieving such level of discrimination. Eventually, AFE transformed general physicochemical knowledge of elements into an OCM-specific one, while active learning enhanced the machine’s accuracy in discriminating elements. The visualization of the feature space is also valuable for uncovering combinatorial rules (Fig. S6). For example, catalysts containing both high-performing Mo and low-performing Pd are found within the cluster of Pd-based catalysts, suggesting that Pd has a more dominant influence than Mo in OCM. Strongly interacting combinations, such as those of Cs with Ti, Zr, and Mo, that are frequently observed in high-performing catalysts, are distributed in small clusters separated from the main cluster for Cs-based catalysts. Additionally, Fe-Zn, while not prominently featured in the training data, is isolated in a very narrow region with relatively high predicted C_2_ yields, an aspect to be explored further.

### Validation, limitations, and future prospects

**Fig. 4:**
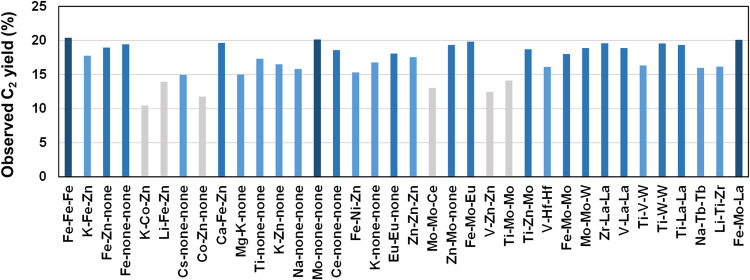
Discovery of high-performing OCM catalysts using the developed ML model. Herein, 36 catalysts were selected from a subset of catalysts with predicted C_2_ yields ≥ 15% using FPS. The bars represent experimentally obtained C_2_ yields, with colors indicating the yield levels.

The primary advantage of AFE, particularly when combined with active learning, lies in its high predictive accuracy and applicability across a wide range of catalysts. To showcase this, we applied FPS to a subset of catalysts with predicted C_2_ yields ≥ 15% using the model obtained after active learning; this resulted in the recommendation of 36 catalysts. Subsequent experimental evaluation revealed that 30 out of the 36 catalysts actually exhibited C_2_ yields ≥ 15%, with 16 of them surpassing a yield of 18% (Fig. 4, Table S6). This is compared to only 37 cases exceeding a yield of 18% among 175 catalysts in the training data. These catalysts predominantly comprise elements whose oxides possess high basicity, such as alkaline, alkaline earth, and rare earth metal elements, along with early transition metal elements from groups 4 to 6. By contrast, many of the high-performing catalysts identified in Fig. 4 do not conform to this pattern, with a notable presence of elements like Fe and Zn. These elements are largely underexplored in the history of OCM research^42^. A unique advantage of our methodology lies in utilizing the integration of AFE and HTE to systematize the model’s education, rather than solely focusing on catalyst discoveries. As a result, the model, enhanced through active learning, significantly streamlined the discovery of high-performing catalysts.

The preceding discussions have elucidated the usefulness of the model involving the engineered features in understanding catalyst design rules and identifying various high-performing catalysts. Conversely, directly extracting physical insights from the engineered features themselves is currently not practical. The engineered features, either individually or in combination, exhibit statistical correlations with catalytic performance. However, statistical correlations do not guarantee causality in catalysis. Moreover, the physical properties of single elements used to generate catalyst features are logically too distant from causal relationships. For instance, the model obtained after active learning is presented as a combination of features: 22.0 (first_ion_en_max)^3^ + 3.32 ln(gs_mag_moment_min)^−1^ − 8.63 (Polarizability_min)^−0.5^ + 4.59 (dipole_polarizability_min)^−0.5^ − 4.22 lattice_constant_min − 6.44 exp(electron_affinity_pro)^−1^ + 10.0 (gs_mag_moment_std)^2^ + 3.26 hhi_r_max + 27.8, among which Polarizability_min, dipole_polarizability_min, and first_ion_en_max are identified to be particularly impactful. These features serve to discriminate between elements whose oxides exhibit strong basicity, those that are useful for O_2_ activation, and other elements, particularly late transition metal elements that catalyze unselective combustion. However, such interpretations are not insights gained directly from the features themselves but rather post hoc explanations assigned to their roles with reference to existing knowledge. Indeed, attempts to extract physical insights based on elemental features have been hardly successful in literature^24,26^. To extract physical insights from the engineered features without relying on prior knowledge, a diverse and comprehensive collection of catalytically relevant properties of elements, so-called a catalysis feature library, is essential (e.g., formation energies of oxides, redox properties, acidity/basicity, and interaction with various molecules). Such a library, albeit currently unavailable, would leverage the advantage of AFE’s compatibility with simple and interpretable ML models. This catalysis feature library, in addition to transparent ML models^43^, is another indispensable piece for achieving fully interpretable catalyst informatics, where density functional theory calculations are expected to play a significant role^44^.

## Conclusion

In summary, we developed and demonstrated AFE as a versatile technique, facilitating effective ML for small datasets of solid catalysts characterized by diverse compositions. AFE exceled in designing highly expressive features tailored to a specific catalyst system without requiring prior knowledge of the system. The availability of process-consistent datasets obtained through HTE was crucial in the development of AFE. The integration of AFE, FPS, and HTE in an iterative loop through active learning systematized the process to educate the machine, promoting the elimination of alternative hypotheses and the identification of a true hypothesis set that applies to a wide array of catalysts. This success can be attributed to the ability of the machine to develop a feature or knowledge space for recognizing the composition–performance relationships of catalysts. Our systematic approach led the enhanced machine to equip remarkable efficiency in pinpointing various high-performing catalysts. However, the extraction of direct insights from engineered features remains a future challenge, necessitating a comprehensive collection of catalytically relevant properties. The integration of AFE into automated experiments^45^ would enable highly efficient autonomous catalyst designs. Furthermore, the knowledge acquired for a specific system is not only beneficial for predicting the performance of unknown compositions within the same system but also for facilitating knowledge acquisition for different systems through transfer learning. As the machine accumulates knowledge across diverse catalytic systems, it is poised to develop comprehensive catalytic knowledge. This advancement promises a future in catalyst development that transcends reliance on researchers’ experiences and knowledge.

## Methods

### Automatic feature engineering

Feature engineering is an essential part of catalyst informatics, as constructing predictive ML models necessitates features that capture the essence of catalysts. Although deep learning can automate feature engineering, the accompanying training requires big data and is often not suitable in the catalysis field where small data are prevalent. Consequently, current feature engineering heavily relies on researchers’ intuition, but this empirical approach is insufficient for exploring diverse designs of catalysts. To address this challenge, we developed an AFE technique capable of handling small data on a variety of materials, including catalysts, without prior knowledge. This technique involves assigning features, synthesizing higher-order features, and selecting important features in the context of supervised ML (Fig. 1). Each step is detailed below, using multi-element solid catalysts as a representative example.

### Feature assignment

A feature library is created by collecting all possible properties of elements from public databases. It can be appropriately normalized and shifted to prevent the divergence of first-order features. Commutative operations are applied to this feature library to assign primary features (denoted as **X**_**0**_) that consider the notational order invariance and elemental composition of individual catalysts^20^. We adopted 58 features of elements stored in XenonPy^33^ and applied eight types of commutative operations (maximum, minimum, weighted sum, weighted average, weighted sum of squared distance, weighted average squared distance, weighted product, and weighted geometric mean), resulting in 464 primary features.

### Feature synthesis

Expressive ML models generally require larger training datasets. Simpler models are suitable for small data, but the reduced expressiveness must be compensated through feature engineering. Therefore, first-order features (f(**X**_**0**_)) that consider nonlinearity and second- or higher-order features (f(**X**_**0**_)·g(**X**_**0**_), etc.) that combines two or more first-order features are synthesized^21–23^. We adopted 12 types of functions (*x*, *x*^1/2^, *x*^2^, *x*^3^, exp(*x*), ln(*x*), and their reciprocals), resulting in 5568 first-order features.

### Feature selection

Identifying a feature subset is crucial for constructing predictive models, as it is not feasible to use all synthesized higher-order features for model fitting. Despite the availability of several feature selection techniques, an exhaustive approach is typically recommended. We employed a genetic algorithm mainly to minimize the MAE value in LOOCV with a specified number of selected features. Huber regression^34^ was adopted owing to its superior performance in handling experimental noise and singular catalysts compared to that of its non-robust counterpart.

AFE was implemented using Python 3.8 and common libraries such as Pandas, NumPy, and scikit-learn, executed in parallel on a PC cluster. The significance of each step is outlined in Table S7, wherein AFE was applied to the OCM dataset, with certain steps intentionally omitted. The analysis revealed that both feature assignment and feature selection were critical for producing a meaningful model, emphasizing the importance of selecting appropriate physicochemical descriptions of catalysts. Higher-order features resulted in a systematic improvement in the score by providing more direct features to the target variable. For small datasets like the OCM dataset, controlling the overfitting in complex models such as random forest regression was difficult. A genetic algorithm (an exhaustive approach) yielded better feature sets than sequential feature selection (a greedy approach)^46^.

### Dataset

We used three HTE datasets for different heterogeneous catalytic systems to demonstrate AFE (Tables S2‒S4). These datasets were obtained using a single protocol, rendering them process-consistent, a crucial feature for reliable ML^47^. A brief overview of the datasets is provided below, with additional details available in published papers^27–32^.

### Dataset for oxidative coupling of methane

The C_2_ yields of 95 M1‒M2‒M3/BaO catalysts during OCM were collected^27–30^. M1‒M3 were selected from Li, Na, Mg, K, Ca, Ti, V, Mn, Fe, Co, Ni, Cu, Zn, Sr, Y, Zr, Mo, Pd, Cs, Ba, La, Ce, Nd, Eu, Tb, Hf, W, and none (blank), with repetitive selection allowed. The amount of each element was fixed at 0.371 mmol per gram support. Although most catalysts were obtained through random selection of elements, certain catalysts were recommended by different ML methods. The experimental protocol used to obtain this dataset is identical to that of the high-throughput experiment described later in this section.

### Dataset for conversion of ethanol to butadiene

The butadiene (C_4_H_6_) yields in ethanol conversion were collected for 177 catalysts^31^. The catalysts were prepared by co-supporting up to 14 elements (Mg, Zn, Cu, Ag, Ni, Al, La, Y, Hf, Zr, Cr, Ga, Nb, and Mo) on SBA-15 through wet impregnation. The loadings of individual elements were optimized within a total loading of 3.00 mmol per gram support to maximize the C_4_H_6_ yield using a genetic algorithm. The C_4_H_6_ yield was measured using a catalyst bed packed in a fused quartz reactor (bed height: 2.0 cm; inner diameter: 4 mm on the influent side and 2 mm on the effluent side) at 400 °C and 21.8 mL min^−1^ of 8.4% ethanol diluted in Ar.

### Dataset for three-way catalysis

The light-off temperatures of 51 nanoparticle-supported catalysts for NO reduction in three-way catalysis were collected^32^. The light-off temperature is defined as the temperature at 50% NO conversion. Bimetallic to pentametallic nanoparticles with equimolar compositions and containing at least one Pt-group element were prepared using a hot-injection method and deposited onto a γ-Al_2_O_3_ support at 0.3 wt%. Temperature ramping experiments were performed using a catalyst bed packed in a fused quartz reactor (bed weight: 60 mg; inner diameter: 4 mm on the influent side and 2 mm on the effluent side) with a 10 mL min^−1^ gas flow of a stoichiometric mixture of CO (13000 ppm), C_3_H_6_ (2000 ppm), NO (3000 ppm), CO_2_ (100000 ppm), O_2_ (14000 ppm), and He (balance).

### High-throughput experiment

To demonstrate active learning, selected catalysts were actually prepared and evaluated using the same experimental method that was used to obtain the training data^27–30^. The catalysts were sampled from a pool of 4060 candidates, generally expressed as M1‒M2‒M3/BaO. M1‒M3 were chosen from Li, Na, Mg, K, Ca, Ti, V, Mn, Fe, Co, Ni, Cu, Zn, Sr, Y, Zr, Mo, Pd, Cs, Ba, La, Ce, Nd, Eu, Tb, Hf, W, or none, with repetitive selection allowed. They were prepared using a parallelized impregnation method using LiNO_3_, NaNO_3_, Mg(NO_3_)_2_, KNO_3_, Ca(NO_3_)_2_·4H_2_O, Ti(O*i*Pr)_4_, VOSO_4_·*x*H_2_O (*x* = 4), Mn(NO_3_)_2_·6H_2_O, Fe(NO_3_)_3_·9H_2_O, Co(NO_3_)_2_·6H_2_O, Ni(NO_3_)_2_·6H_2_O, Cu(NO_3_)_2_·3H_2_O, Zn(NO_3_)_2_·6H_2_O, Sr(NO_3_)_2_, Y(NO_3_)_3_·6H_2_O, ZrO(NO_3_)_2_·2H_2_O, (NH_4_)_6_Mo_7_O_24_·4H_2_O, Pd(OAc)_2_, CsNO_3_, Ba(NO_3_)_2_, La(NO_3_)_3_·6H_2_O, Ce(NO_3_)_3_·6H_2_O, Nd(NO_3_)_3_·6H_2_O, Eu(OAc)_3_·4H_2_O, Tb(NO_3_)_3_·5H_2_O, Hf(OEt)_4_, and (NH_4_)_10_H_2_(W_2_O_7_)_6_ as precursors. These precursors were obtained from Sigma-Aldrich, Kanto Chemical, Wako Pure Chemical Industries, and Alfa Aesar. Ba(OH)_2_·8H_2_O purchased from Wako Pure Chemical Industries was used as the precursor for the BaO support. The support powder (1.0 g) was suspended in 4‒5 mL of a precursor solution under stirring at 50 °C for 6 h. The concentration of the solution was adjusted to 0.371 mmol per gram support for each of the selected elements. After drying, the catalyst was calcined in air at 1000 °C for 3 h and thoroughly ground using a mortar and pestle before use. When using metal alkoxides, impregnation was performed in two steps, starting with an aqueous solution and followed by an ethanol solution of the metal alkoxides.

The performance of the catalysts in OCM was evaluated using an in-house high-throughput screening instrument^47^. The instrument comprises a gas mixer for generating the reaction gas mixture (MU-3504, HORIBA STEC), a gas distributor for splitting the reaction gas equally into 20 reactor tubes (fused quartz tubes with an inner diameter of 4 mm on the influent side and 2 mm on the effluent side) loaded with catalyst powder and symmetrically placed in a hollow electric furnace, and an auto-sampler for supplying the effluent gas from individual tubes to a quadruple mass spectrometer (Transpector CPM 3, INFICON). Mass signals were converted into the relative pressures of individual gases based on external calibration. Cooperative action among the programmed gas generation, temperature, and auto-sampling enabled an automatic evaluation of the performance of 20 catalysts under a predetermined set of reaction conditions.

The catalyst powder was packed at a height of 10 mm in the neck of the reactor tube using quartz wool and was in-line calcined at 1000 °C under an O_2_ atmosphere for 3 h. A reaction gas mixture of CH_4_ and O_2_ balanced with Ar was flowed through the 20 tubes, and the temperature was decreased stepwise from 900 to 700 °C in 50 °C increments. The total gas flow volume (10, 15, and 20 mL min^−1^), CH_4_/O_2_ ratio (2, 4, and 6 mol mol^−1^), and Ar concentration (P_Ar_ = 0.15, 0.40, and 0.70 atm) were respectively varied at each temperature, resulting in a total of 135 reaction conditions. The C_2_ yield, defined as the percentage of the doubled sum of the partial pressures of C_2_H_6_ and C_2_H_4_ relative to that of CH_4_ in the influent, was obtained at each of the 135 conditions, and the maximum C_2_ yield was recorded for further analysis.

### Supplementary information

Summplementary Information

## Data Availability

The three datasets used to demonstrate automatic feature engineering in Fig. 1 are curated from published papers and listed in the Supplementary Information. The authors declare that all data supporting the findings and those used for reproducing the figures in this paper are available within the paper and its Supplementary Information. Source data are provided with this paper.

## Code availability

Codes are available at https://github.com/TaniikeLaboratory/Automatic-feature-engineering-for-catalyst-small-data.
