# Supplementary material for: Automatic feature engineering for catalyst design using small data without prior knowledge of target catalysis
Source: Commun Chem. 2024 Jan 12;7:11. doi: 10.1038/s42004-023-01086-y (PMC10786848; doi:10.1038/s42004-023-01086-y)
Supplement: Supplementary file 1 — Summplementary Information [file 42004_2023_1086_MOESM1_ESM.pdf]

Supplementary Information

Automatic feature engineering for catalyst design using small data without prior knowledge of target catalysis

Toshiaki Taniike[1*], Aya Fujiwara[1], Sunao Nakanowatari[1], Fernando García-Escobar[2], and Keisuke Takahashi[2]

[1]Graduate School of Advanced Science and Technology, Japan Advanced Institute of Science and Technology, 1-1 Asahidai, Nomi, Ishikawa 923-1292, Japan

[2]Department of Chemistry, Hokkaido University, North 10, West 8, Sapporo 060-0810, Japan

[*]Corresponding author: e-mail: taniike@jaist.ac.jp

Table S1: Definition of features

Feature type	General formula
Primary[a]	$X_0^{i,j} = o_j\left(x_1^i, x_2^i, \dots, x_n^i, w_1, w_2, \dots, w_n\right)$
First order[b]	$X_1^{i,j,k} = f_k\left(X_0^{i,j}\right)$
Second order[c]	$X_2^{i1,j1,k1,i2,j2,k2} = X_1^{i1,j1,k1} \cdot X_1^{i2,j2,k2}$
Third order[c]	$X_3^{i1,j1,k1,i2,j2,k2,i3,j3,k3} = X_1^{i1,j1,k1} \cdot X_1^{i2,j2,k2} \cdot X_1^{i3,j3,k3}$

[a] A primary feature, $X_0^{i,j}$, refers to a feature obtained by applying a commutative operation, o_j, to a set of values, $x_1^i, x_2^i, \dots, x_n^i, w_1, w_2, \dots, w_n$, where x_1^i and w_1 correspond to the value of the *i*-th feature (e.g., electronegativity) and quantity of component 1 in a catalyst. The commutative operations employed in this study were the maximum, minimum, weighted sum, weighted average, weighted sum of squared distance, weighted average squared distance, weighted product, and weighted geometric mean; [b] A first-order feature, $X_1^{i,j,k}$, is obtained by applying a function, f_k, to a primary feature, $X_0^{i,j}$. Twelve types of functions (x, $x^{1/2}$, x^2, x^3, exp(x), ln(x), and their reciprocals) were employed in this study; [c] Second- and higher-order features are the products of two or more first-order features.

Table S2: Dataset for the oxidative coupling of methane[a]

No.	M1–M2–M3[b]	Observed C_2 yield[c] (%)	Predicted C_2 yield[d] (%)
1	Li–Mg–Zr	18.6	17.5
2	Li–Co–Zn	6.2	9.4
3	Li–Co–Nd	7.1	7.1
4	Li–Co–Eu	13.8	7.4
5	Li–Zr–Cs	16.4	16.3
6	Na–Ca–Mn	9.0	8.8
7	Na–Fe–Ce	9.3	12.1
8	Mg–K–Y	17.0	17.0
9	Mg–V–Mn	10.2	11.0
10	Mg–Mn–Ni	8.7	6.6
11	Mg–Pd	0.8	1.1
12	Mg–Ni–W	9.6	5.9
13	K–Ca–Zr	13.7	15.6
14	K–V–Mo	18.5	17.7
15	K–Zr–La	17.5	15.9
16	Ca–Mn–Mo	10.3	10.4
17	Ca–Y–Zr	11.0	11.0
18	Ca–W	6.2	14.7
19	V–Mn–Cu	10.0	9.1
20	V–Fe	13.9	17.8
21	V–Zr–Eu	12.9	14.2
22	Mn–Y–Hf	6.1	9.4
23	Fe–Ba–La	14.1	14.1
24	Co–Zn–Zr	12.4	12.4
25	Zn–Hf	16.9	17.6
26	Sr–Mo	21.2	18.7
27	Sr–Ba–Hf	10.6	13.6
28	Mo–Cs–W	20.2	19.2
29	Ce–Nd–Hf	15.2	15.2
30	Fe–Fe–W	16.5	16.6
31	Mg–Mo–Hf	18.7	16.6
32	K–Mo–La	3.3	17.5
33	Fe–Ce	15.8	15.1

No.	M1–M2–M3[b]	Observed C_2 yield[c] (%)	Predicted C_2 yield[d] (%)
34	Li–Zr–Mo	13.8	18.1
35	Na–Sr–Mo	17.4	17.6
36	Li–Mo–La	18.0	17.5
37	Li–Mo–Nd	15.9	17.5
38	Li–Mo–Ba	20.2	17.2
39	Li–Ba–Nd	17.2	17.2
40	Li–Nd–Tb	17.5	16.3
41	Li–Y–Eu	15.3	16.7
42	K–Ti–W	16.5	16.4
43	Ti–Cs–W	17.5	16.1
44	Ti–Tb–W	17.1	16.2
45	Sr–Hf	15.0	13.8
46	V–Sr	11.8	14.0
47	Sr–Mo–Hf	13.3	16.6
48	Sr–Mo–W	13.5	17.5
49	Sr–Mo–Ba	16.8	16.7
50	Li–Mo–Cs	17.4	18.1
51	Li–Mo–W	16.3	18.5
52	V–Mo–W	14.3	17.2
53	K–Mo–W	18.4	18.4
54	Zr–Mo–Cs	18.0	18.8
55	Zr–Cs–W	17.3	16.2
56	K–V–W	15.0	15.4
57	K–Y–Mo	17.6	18.1
58	K–V–Y	18.2	14.7
59	Mg–Zr–Eu	18.8	18.2
60	K–V–Eu	20.4	14.1
61	V–Mo–Eu	17.0	16.7
62	K–Ca–Mo	18.2	17.3
63	K–V–Zr	14.8	14.9
64	Mg–Zr–Cs	15.2	16.9
65	Mg–Y–Zr	18.6	18.5
66	Li–Ti–W	19.0	16.8
67	Mg–Ca–Sr	17.4	17.0

No.	M1–M2–M3[b]	Observed C_2 yield[c] (%)	Predicted C_2 yield[d] (%)
68	Mg–K–Ba	12.8	15.6
69	Eu–Hf–W	16.9	16.9
70	Fe–Mo–Hf	16.0	15.4
71	Cu–Cu–La	2.4	8.0
72	Li–Zn–Tb	14.8	14.5
73	Li–K–Fe	12.5	12.2
74	V–Co–Cs	10.3	10.3
75	Ti–Mo–Cs	17.7	18.5
76	Na–Ni–W	0.0	4.1
77	Eu–Eu–W	16.2	16.9
78	Ti–Cs–Cs	19.6	19.6
79	Mg–Pd–Ce	0.0	-0.1
80	Li–Mg–Cs	17.6	16.7
81	Na–Mo–Cs	18.5	18.8
82	K–V–Ba	14.7	13.6
83	K–Mo–Mo	20.6	20.2
84	Sr–Zr–La	22.2	10.8
85	Ca–Pd–Nd	0.0	0.0
86	Cu–Cs–Nd	7.3	7.4
87	Li–Ce–Nd	11.9	17.6
88	Cs–Eu	17.8	18.3
89	Eu–W	16.9	17.4
90	K–Ti–Cu	7.7	7.7
91	Li–Zn–Zn	17.7	16.0
92	Zr–Mo–Eu	17.6	17.7
93	K–La–W	18.4	15.7
94	Cs–Eu–W	17.6	16.8
95	Ce–Hf–W	17.7	16.3

[a] The data were curated from previous studies[1–4]; [b] Catalysts are generally denoted as M1–M2–M3/BaO, where M1–M3 are co-supported on BaO at a loading of 0.371 mmol per gram support; [c] Maximum C_2 yield observed among the 135 reaction conditions; [d] Obtained using a Huber regressor with eight selected first-order features.

Table S3: Dataset for the conversion of ethanol to butadiene[a]

No.	Composition[b] (mmol g-support^{-1})														Observed C_4H_6 yield[c] (%)	Predicted C_4H_6 yield[d] (%)
	Mg	Zn	Cu	Ag	Ni	Al	La	Y	Hf	Zr	Cr	Ga	Nb	Mo		
1	0	0.1107	0.1599	0.0369	0.3075	0.0123	0.0246	0.0492	0.0123	0.1353	0.1353	0.0984	0.1353	0.0123	30	27
2	0.0099	0	0.1188	0.099	0.0396	0	0.1188	0.0693	0.1683	0.0099	0.198	0.1089	0.0198	0.0297	26	19
3	0	0.054	0.0702	0	0.0432	0.0054	0.0216	0.0378	0.0054	0.1782	0.0648	0.0108	0.0378	0.0108	32	31
4	0.1323	0.2205	0.0882	0.3822	0.1323	0.0294	0.0294	0.0441	0	0.1029	0.0441	0.0441	0.147	0.0735	34	34
5	0.042	0.1008	0.1008	0.0336	0.1008	0	0.0168	0.0084	0	0.1176	0.1092	0.0672	0.1428	0	28	32
6	0.1281	0.0732	0.6039	0.2379	0.1098	0.1098	0.2013	0	0	0.0366	0.1281	0.1281	0.0366	0.0366	12	11
7	0.0204	0.0153	0.0816	0.0153	0.0255	0.0051	0	0.051	0.0153	0.051	0.0714	0.0561	0.102	0	12	10
8	0.0369	0.0615	0.1476	0.3075	0.0615	0.0738	0.0123	0.2583	0.0123	0.0246	0.0492	0.0492	0	0.1353	13	21
9	0.1128	0.0987	0.0705	0.0141	0.282	0	0.0987	0.0987	0.0846	0.1974	0.0705	0.141	0.0846	0.0564	33	33
10	0	0	0.2844	0.4503	0.2133	0.0237	0.6636	0.1659	0.1896	0	0.1896	0.1185	0	0.0711	21	29
11	0	0.1215	0.018	0	0.0045	0.054	0.0405	0	0.027	0.0045	0.054	0.018	0.036	0.072	20	20
12	0.2016	0.0288	0.0384	0.1824	0.1152	0.0288	0.0864	0.1728	0.0288	0.0096	0	0	0.0672	0	25	24
13	0.0621	0.0828	0.3105	0.4347	0.0828	0.0207	0.0621	0.207	0.0414	0.3519	0	0.2691	0.1449	0	28	27
14	0	0	0.066	0	0.0528	0.132	0.1716	0	0.2376	0.0396	0.0528	0.198	0.1584	0.2112	15	14
15	0.0318	0.0954	0.0954	0.1431	0.0795	0.0477	0.0159	0.3498	0.0636	0.0795	0.0159	0.0318	0.2703	0.2703	21	26
16	0.432	0.024	0.024	0.048	0.096	0.024	0.192	0.144	0.6	0.072	0.264	0.288	0	0.192	23	23
17	0.0336	0.0252	0.3024	0.0924	0.0168	0.084	0	0.1176	0	0.0336	0.0588	0	0	0.0756	8	8
18	0.099	0.0792	0.1386	0.0396	0.1584	0.4752	0.0198	0.1188	0.1782	0.3564	0.0792	0.0792	0.0198	0.1386	28	19
19	0.0111	0.0777	0.111	0.0777	0.0888	0.0444	0.0777	0.0444	0.1665	0.0777	0.0333	0.1332	0.0555	0.0999	33	30

No.	Composition[b] (mmol g-support^{-1})														Observed C_4H_6 yield[c] (%)	Predicted C_4H_6 yield[d] (%)
	Mg	Zn	Cu	Ag	Ni	Al	La	Y	Hf	Zr	Cr	Ga	Nb	Mo		
20	0.0141	0.0141	0.0141	0.0846	0.0564	0.1692	0.1692	0.1692	0.0705	0.1551	0.0564	0.1269	0.1692	0.1269	14	16
21	0.0615	0.1476	0.1476	0.0492	0.1476	0.0123	0.0246	0.0123	0.0123	0.1353	0.1353	0.0984	0.2091	0.0369	30	31
22	0.1035	0.0828	0	0.0414	0.0828	0.4968	0.0207	0.207	0.0414	0.3519	0.0828	0.2691	0.1449	0.1449	15	15
23	0.0528	0.0396	0.2904	0.1452	0.0264	0.132	0	0.1848	0.2376	0.0396	0.0528	0	0	0.1188	20	20
24	0.0099	0.0099	0.198	0.0396	0.0396	0	0.1188	0.099	0.0891	0.0099	0.198	0.0198	0.0198	0.1386	12	16
25	0	0.0924	0.066	0.0132	0.0528	0.132	0.1716	0.0924	0.0792	0.1848	0.066	0.198	0.0792	0.0924	27	26
26	0.0318	0.0954	0.0954	0.4134	0.0795	0.0477	0.0159	0.0795	0.0636	0.0795	0.0159	0.0318	0.2703	0.2703	23	23
27	0	0	0.1008	0.0336	0.42	0.0084	0.0168	0.0588	0.0672	0	0.0672	0.042	0	0.0252	10	10
28	0	0.1107	0.246	0.0492	0	0.0123	0.123	0.123	0.0123	0.2214	0	0.0246	0.1353	0.1722	36	36
29	0	0.054	0.108	0	0.0432	0	0.054	0.054	0.0054	0.1296	0.0648	0.0108	0.0054	0.0108	29	30
30	0	0.054	0.0702	0	0.0432	0.0054	0.0216	0.0378	0.0054	0.1782	0.0648	0.0108	0.0378	0.0108	32	31
31	0.0432	0.054	0.0702	0.0054	0	0.0054	0.0162	0.0378	0.0054	0.1782	0.0648	0.0108	0.0378	0.0108	34	34
32	0.1323	0.0735	0.0882	0.3822	0.1323	0.0294	0.0294	0	0.0882	0.2058	0.0441	0.0441	0.147	0.0735	28	31
33	0.021	0.0945	0.1365	0.0315	0.2625	0.0105	0	0.042	0.0105	0.1155	0.1155	0.084	0.1155	0.0105	27	25
34	0	0.0102	0.1326	0.0306	0.255	0.0918	0.0204	0.0408	0.0102	0.1122	0.1122	0.0816	0.1122	0.0102	10	11
35	0.1272	0.0159	0.0954	0.1908	0	0.0477	0.0795	0.0477	0.2544	0.2862	0.2385	0	0.2067	0	26	23
36	0.0561	0.0612	0.0051	0.051	0.0714	0.0612	0.0153	0.0153	0	0.0102	0.0663	0	0.0714	0.0255	13	16
37	0	0.054	0.0702	0	0.0432	0.0054	0.0216	0.0378	0.0054	0.1782	0.0648	0.0108	0.0378	0.0108	32	31
38	0.1323	0.2205	0.0882	0.3822	0.1323	0.0294	0.0294	0.0441	0	0.1029	0.0441	0.0441	0.147	0.0735	31	34
39	0.0486	0.054	0.0702	0.1188	0.0432	0	0	0.0378	0	0.0378	0.0648	0	0.0378	0.027	18	25

No.	Composition[b] (mmol g-support^{-1})														Observed C_4H_6 yield[c] (%)	Predicted C_4H_6 yield[d] (%)
	Mg	Zn	Cu	Ag	Ni	Al	La	Y	Hf	Zr	Cr	Ga	Nb	Mo		
40	0	0.054	0.0702	0	0.0432	0.0054	0.0216	0.0378	0.0054	0.1782	0.0648	0.0108	0.0378	0.0108	30	31
41	0.0105	0.0735	0.1365	0.0315	0.084	0.042	0.0735	0.042	0.252	0.0735	0.0315	0.126	0.0525	0.0105	39	37
42	0.084	0.0945	0.0525	0.0105	0.21	0.0105	0.0735	0.0735	0.063	0.1155	0.0525	0.105	0.063	0.042	34	33
43	0.1323	0.2205	0.0882	0.3822	0.1323	0.0147	0.0294	0.0441	0	0.1029	0.1029	0.0441	0.1029	0.0735	38	34
44	0.1323	0.2205	0.0735	0	0	0.0294	0.1911	0.1029	0	0.2058	0.0441	0.2205	0.147	0.1029	45	36
45	0	0.054	0.0702	0	0.0432	0.0054	0.0216	0.0378	0.0054	0.1782	0.0648	0.0108	0.0378	0.0108	38	31
46	0.1323	0.0735	0.147	0.3822	0.1323	0.0294	0.0294	0	0.0882	0.2058	0.0441	0.0588	0.0735	0.0735	32	30
47	0	0.054	0.0702	0.0162	0.0432	0.0054	0.0216	0.054	0.0054	0.1782	0.0648	0.0108	0.0054	0.0108	34	32
48	0	0.0861	0.0615	0.0123	0.246	0.0123	0.0246	0.0861	0.0738	0.1722	0.2337	0.0984	0.0738	0.0492	39	29
49	0	0.1107	0.246	0.0492	0.0123	0.0123	0.123	0.123	0	0.2214	0	0.0246	0.1353	0.1722	43	31
50	0.0123	0.1107	0.246	0.0492	0	0	0.123	0.123	0.0123	0.2214	0	0.0246	0.1353	0.1722	37	37
51	0.0357	0.0408	0.0408	0.0612	0.0408	0.0204	0.0612	0.0306	0.0102	0.0612	0.0459	0.0051	0.0561	0.0051	13	22
52	0.0384	0.1344	0.2112	0.1344	0.1536	0.288	0.2112	0.096	0.1536	0.2688	0	0.1344	0.0384	0.0768	27	28
53	0.1323	0.2205	0.0735	0	0.1764	0.0294	0.1911	0.1029	0	0.2058	0.0441	0.0441	0.147	0.1029	37	36
54	0.1323	0.2205	0.0882	0.3822	0.1323	0.0147	0.0294	0.0441	0	0.1029	0.1029	0.0441	0.1029	0.0735	36	34
55	0	0.1107	0.246	0.0492	0.0123	0.0615	0.123	0.123	0	0.1722	0	0.0246	0.1353	0.1722	29	28
56	0.0162	0.054	0.0702	0.0162	0.0432	0.0054	0.0216	0.0378	0.0054	0.1782	0.0648	0.0108	0.0054	0.0108	33	32
57	0.1323	0.2205	0.0735	0	0	0.0294	0.1911	0.1029	0	0.2058	0.0441	0.0294	0.147	0.294	36	34
58	0.0105	0.0735	0.0735	0.042	0.084	0.042	0.0735	0.105	0.252	0.0735	0.0315	0.126	0.0525	0.0105	39	38
59	0.1323	0.2205	0.0735	0	0	0.0294	0.1911	0.1029	0	0.2058	0.0441	0.2205	0.147	0.1029	36	36

No.	Composition[b] (mmol g-support^{-1})														Observed C_4H_6 yield[c] (%)	Predicted C_4H_6 yield[d] (%)
	Mg	Zn	Cu	Ag	Ni	Al	La	Y	Hf	Zr	Cr	Ga	Nb	Mo		
60	0.1323	0	0.1911	0.3822	0.1176	0.0294	0.0294	0.1029	0.0294	0.2058	0.0441	0.0294	0.1029	0.0735	15	17
61	0.1323	0.2205	0.0882	0.0588	0.1764	0.0147	0.147	0.147	0	0.1029	0	0.0294	0.147	0.2058	32	33
62	0	0.0612	0.0765	0.0204	0	0	0.051	0.051	0.0051	0.0918	0.0663	0.0102	0.0561	0.0255	36	30
63	0	0.0861	0.123	0.0861	0	0.0492	0.0738	0.0492	0.1845	0.0861	0.0369	0.1476	0.1353	0.1722	27	31
64	0	0.1107	0.246	0.0492	0.0123	0.0123	0.123	0.123	0	0.2214	0	0.0246	0.1353	0.1722	32	31
65	0.1323	0.2205	0.0735	0.0441	0	0.0294	0.1911	0.1029	0	0.2058	0	0.2205	0.147	0.1029	36	37
66	0.1323	0.2205	0.0735	0	0.1029	0.0294	0.1911	0.1029	0	0.2058	0.0441	0.2205	0.147	0	38	36
67	0.0336	0.0624	0.0336	0.0096	0.0288	0.048	0.0432	0.0624	0.0576	0.0048	0.0288	0.0096	0.0432	0.0096	28	26
68	0.126	0.063	0.054	0.063	0.126	0.081	0.036	0.027	0.009	0.081	0.036	0.081	0.081	0.027	22	22
69	0	0.1107	0.246	0.0492	0.0123	0.0123	0.123	0.123	0	0.2214	0	0.0246	0.1353	0.1722	33	31
70	0.1107	0.1845	0.0615	0.0492	0.1476	0.0123	0.123	0.0861	0	0.1722	0.0369	0.0246	0.1353	0.0861	40	35
71	0.0123	0.0615	0.0861	0.0861	0.0984	0.0492	0.0738	0.0492	0.2952	0.0861	0.0369	0.1476	0.1353	0.0123	34	34
72	0.0123	0.0861	0.246	0.0492	0	0.0123	0.123	0.123	0.0615	0.2214	0.0369	0.0246	0.0615	0.1722	34	35
73	0.0441	0.2205	0.0735	0.0735	0.1176	0.0294	0.0588	0.1029	0.0147	0.4851	0.1764	0.0294	0.0147	0.0294	42	43
74	0	0.1575	0.0735	0.042	0	0.042	0.1365	0.105	0.0105	0.147	0.0315	0.126	0.105	0.0735	36	35
75	0.1323	0.147	0.0882	0.3822	0.1323	0	0.0294	0.147	0	0.1029	0.1029	0.0294	0.1029	0.0735	32	32
76	0	0.1845	0.0615	0.0369	0.0246	0.0246	0.1599	0	0.0738	0.1722	0.2337	0.0984	0.0738	0.0861	32	32
77	0.0105	0.0735	0.0735	0.042	0.084	0.042	0.0735	0.042	0.252	0.0735	0.0315	0.189	0.0525	0.0105	36	36
78	0.1323	0.2205	0.0735	0.0588	0	0.0294	0.1911	0.1029	0.0588	0.2058	0.0441	0.1764	0.0735	0.1029	39	39
79	0.0162	0.054	0.0702	0.0162	0.0432	0.0054	0.0216	0.0378	0	0.1782	0.0648	0.0108	0.0054	0.0162	32	31

No.	Composition[b] (mmol g-support^{-1})														Observed C_4H_6 yield[c] (%)	Predicted C_4H_6 yield[d] (%)
	Mg	Zn	Cu	Ag	Ni	Al	La	Y	Hf	Zr	Cr	Ga	Nb	Mo		
80	0	0.0735	0.0735	0.042	0.084	0.042	0.0735	0.105	0.252	0.189	0.0315	0.021	0.0525	0.0105	40	41
81	0.1323	0.2205	0.0735	0	0	0.0294	0.1911	0.1029	0	0.0441	0.2058	0.2205	0.147	0.1029	22	31
82	0.1323	0.2205	0.0735	0	0	0.0294	0.1911	0.1029	0	0.2058	0.0441	0.2205	0.147	0.1029	34	36
83	0.132	0.264	0.2112	0.3168	0.1584	0.264	0.1584	0.1584	0.0528	0.132	0.2112	0.2376	0.2376	0.132	23	18
84	0.0546	0.0078	0.0468	0.0195	0.0195	0.0117	0.0507	0.0195	0.039	0.039	0.0312	0.0039	0.039	0.0117	12	15
85	0.0945	0.0735	0.0945	0	0.084	0.042	0.1365	0.0735	0	0.147	0.0315	0.1575	0.105	0.0105	30	27
86	0.0105	0.0735	0.0735	0.042	0.084	0.042	0.0735	0.105	0.252	0.0735	0.0315	0.126	0.0525	0.0105	34	38
87	0	0.1107	0.0861	0.0861	0.0123	0.0492	0.0738	0.0492	0	0.0861	0	0.5289	0.1353	0.0123	18	21
88	0	0.1107	0.246	0	0.0123	0.0123	0.123	0.123	0.0492	0.2214	0	0.0246	0.1353	0.1722	29	33
89	0.1107	0.1845	0.0615	0.0492	0.0123	0.0246	0.1599	0.0861	0	0.2214	0.0369	0.0246	0.0861	0.1722	33	33
90	0.0369	0.1845	0.0615	0.0615	0.0246	0.0246	0.0492	0.0984	0.0738	0.4059	0.1476	0.0246	0.0123	0.0246	40	44
91	0.1323	0.2205	0.0735	0	0	0.0294	0.1911	0.1029	0	0.4263	0.0441	0	0.147	0.1029	31	40
92	0.1107	0.1107	0.1353	0.0492	0.1476	0.0123	0.123	0.0861	0	0.1722	0.0369	0.0246	0.1353	0.0861	29	31
93	0.0105	0.1575	0.0735	0.0315	0.0735	0.021	0.0735	0.042	0.063	0.147	0.1995	0.084	0.063	0.0105	31	34
94	0.1323	0.2205	0.0735	0.0588	0.0147	0.0294	0.1911	0.2205	0	0.2058	0.0441	0.0294	0.147	0.1029	31	35
95	0	0.1107	0.246	0.0492	0.0123	0	0.123	0.123	0.1353	0.2214	0.0369	0.0246	0.1353	0.0123	36	40
96	0	0.1575	0.0735	0.042	0.084	0.042	0.1365	0.021	0.0105	0.147	0.0315	0.126	0.105	0.0735	16	31
97	0.1029	0.2205	0.0735	0	0	0.0294	0.1911	0.1029	0	0.2058	0.0441	0.2205	0.147	0.1323	27	35
98	0.1323	0.2205	0.0735	0	0.1029	0.0294	0.1911	0	0	0.2058	0.0441	0.2205	0.147	0.1029	24	32
99	0.081	0.27	0.135	0.189	0.243	0.27	0.108	0.054	0.27	0.243	0.135	0.27	0.162	0.243	26	23

No.	Composition[b] (mmol g-support^{-1})														Observed C_4H_6 yield[c] (%)	Predicted C_4H_6 yield[d] (%)
	Mg	Zn	Cu	Ag	Ni	Al	La	Y	Hf	Zr	Cr	Ga	Nb	Mo		
100	0.0111	0.0999	0.0444	0.0333	0.0999	0	0.111	0.0999	0.0777	0.0999	0.111	0.111	0.1332	0.0999	29	29
101	0	0.1845	0.0615	0.0861	0	0.0492	0.0738	0.0492	0	0.0246	0.0369	0.5289	0.123	0.0123	15	26
102	0.1107	0	0.1353	0.0492	0.1476	0.0123	0.0861	0.0861	0.2214	0.1722	0.0369	0.0246	0.0615	0.0861	20	23
103	0.0123	0.1107	0.0861	0.0492	0.0984	0.0123	0.0861	0.123	0.4305	0.0861	0.0369	0.0246	0.0615	0.0123	34	49
104	0	0.1107	0.246	0.0861	0.0123	0.0123	0.0738	0.0492	0.2214	0.0861	0	0.0246	0.1353	0.1722	44	36
105	0.0105	0.1575	0.0525	0	0.084	0.021	0.1365	0.0735	0	0.147	0.0315	0.1575	0.105	0.0735	31	32
106	0.0615	0.123	0.0984	0.0492	0.0738	0.123	0.0738	0.0738	0.0246	0.0615	0.0984	0.1107	0.1968	0.0615	17	23
107	0	0.0945	0.21	0.0315	0.0105	0.0105	0.105	0.042	0.063	0.189	0	0.021	0.063	0.21	31	31
108	0.0315	0.1575	0.0735	0.0525	0.084	0.021	0.042	0.0735	0.0105	0.3465	0.105	0.021	0.0105	0.021	25	42
109	0	0.1575	0.0735	0.042	0.084	0.021	0.0735	0.105	0.252	0.147	0.0105	0.021	0.0525	0.0105	51	50
110	0.1107	0.1845	0.0738	0.0492	0	0.0246	0.1599	0.0861	0	0.2214	0.0369	0.0246	0.0861	0.1722	33	36
111	0.0369	0.1107	0.246	0.0615	0.0246	0.0246	0.0492	0.1722	0.0738	0.2214	0.1476	0.0246	0.0123	0.0246	34	35
112	0.0441	0.3822	0.1029	0.0588	0.1176	0.0588	0.1029	0.147	0.0147	0.2646	0.0441	0.0294	0.0735	0.0294	44	44
113	0.1323	0.2205	0.0735	0.0294	0	0	0.1911	0.1029	0	0.2058	0.0441	0.2205	0.147	0.1029	35	37
114	0.1323	0.2205	0.0294	0	0	0.0735	0.1911	0.1029	0	0.2058	0.0441	0.2205	0.147	0.1029	28	34
115	0.0765	0.102	0.2295	0.1275	0.255	0.153	0.204	0.204	0.1785	0.1785	0.204	0.2295	0.1785	0.1785	18	20
116	0.078	0.1092	0.078	0.1092	0.1248	0.1404	0.1248	0.078	0.1092	0.0468	0.1872	0.0624	0.1404	0.1872	52	21
117	0.1107	0.1845	0.246	0.0492	0	0.0123	0.123	0.123	0	0.2214	0	0.0246	0.1353	0	51	43
118	0.0369	0.2829	0.0861	0.0492	0.0984	0.0123	0.123	0.123	0.0123	0.2214	0	0.0246	0.1353	0.0246	21	44
119	0.0111	0.1665	0.0555	0.0777	0	0.0444	0.0666	0.0444	0	0.0444	0	0.4773	0.111	0.0111	48	27

No.	Composition[b] (mmol g-support^{-1})														Observed C_4H_6 yield[c] (%)	Predicted C_4H_6 yield[d] (%)
	Mg	Zn	Cu	Ag	Ni	Al	La	Y	Hf	Zr	Cr	Ga	Nb	Mo		
120	0.0111	0.1665	0.0444	0.0333	0.0888	0	0.222	0.0999	0.0666	0.0999	0.0111	0.111	0.0555	0.0999	43	39
121	0	0.0861	0.0861	0.0492	0.0984	0.0492	0.0861	0.123	0.2952	0.2214	0.0369	0.0246	0.0615	0.0123	14	42
122	0.0105	0.1575	0.0735	0.042	0.084	0.021	0.0735	0.105	0	0.147	0.0315	0.021	0.0525	0.231	29	30
123	0.0945	0.1575	0.0525	0.042	0	0.021	0.1365	0.0315	0	0.147	0.0315	0.1575	0.105	0.0735	33	33
124	0.1323	0.2205	0.0735	0	0	0.0294	0.1911	0.1029	0	0.2058	0.0441	0.2205	0.147	0.1029	34	36
125	0	0	0.21	0.0735	0	0.0315	0.0735	0.042	0.252	0.0735	0.0315	0	0.1155	0.147	18	21
126	0.1323	0.2205	0.0735	0	0	0	0.0294	0.1029	0	0.2058	0.0441	0.2205	0.147	0.294	23	34
127	0.0945	0.1575	0.084	0	0.084	0.021	0.0735	0.105	0.189	0.147	0.0105	0.021	0.0525	0.0105	53	46
128	0	0.1575	0.0735	0.042	0.084	0.021	0.0735	0.105	0.147	0.252	0.0105	0.021	0.0525	0.0105	49	47
129	0	0.1575	0.0735	0.042	0.0105	0.021	0.0735	0.105	0.252	0.147	0.0105	0.021	0.0525	0.084	44	46
130	0.27	0.189	0.081	0.162	0.135	0.189	0.216	0.27	0.216	0.135	0.297	0.297	0.027	0.27	29	22
131	0.0126	0.0036	0.0126	0.009	0.0144	0.0216	0.0198	0.0252	0.018	0.009	0.0108	0.0162	0	0.0108	10	14
132	0	0.1575	0.0735	0.042	0.084	0.021	0.0735	0.105	0.252	0.147	0.0105	0.021	0.0525	0.0105	49	50
133	0	0.1575	0	0.042	0	0	0	0	0.147	0.252	0.0105	0.3255	0.105	0.0105	38	38
134	0.0147	0.2205	0.0735	0.0441	0.1176	0.0294	0.294	0.1323	0.0882	0.1323	0.0147	0.0294	0.147	0.1323	42	40
135	0.0945	0.1575	0.0735	0.042	0.084	0.0315	0.0735	0.105	0.147	0.147	0.0105	0.021	0.0525	0.0105	48	44
136	0.078	0.234	0.078	0	0.1248	0.0312	0.1092	0.078	0.1092	0.0468	0.1872	0.0312	0.4368	0.0156	32	35
137	0.0525	0.1575	0.0735	0.042	0.084	0.0105	0	0.105	0.252	0.147	0.0105	0	0.1155	0	47	49
138	0.0525	0.0735	0.0525	0	0.0105	0.0945	0.084	0.105	0.0735	0.252	0.126	0.021	0.0945	0.0105	25	25
139	0.078	0.1092	0.312	0.1092	0.1248	0	0.1092	0.0624	0.1092	0.0468	0.1872	0.0624	0.1716	0.0936	28	25

No.	Composition[b] (mmol g-support^{-1})														Observed C_4H_6 yield[c] (%)	Predicted C_4H_6 yield[d] (%)
	Mg	Zn	Cu	Ag	Ni	Al	La	Y	Hf	Zr	Cr	Ga	Nb	Mo		
140	0	0.0555	0.0777	0.0444	0.0888	0.0222	0.0777	0.0999	0.2664	0.0999	0.0111	0.111	0.0555	0.0999	27	34
141	0.1107	0.1845	0.246	0.0369	0	0.0123	0.123	0.123	0	0.2214	0	0.0246	0.1353	0.0123	47	42
142	0.1323	0.2205	0.1029	0	0	0.0294	0.1911	0.0294	0.3528	0.2058	0.0441	0	0.147	0.0147	51	50
143	0	0.1575	0.0735	0	0.084	0	0.0735	0.105	0	0.147	0	0.126	0.0525	0.231	30	30
144	0.021	0.1575	0.084	0	0.084	0.021	0.0735	0.105	0.189	0.147	0.0105	0.0945	0.0525	0.0105	43	46
145	0.0945	0.1575	0.084	0	0.084	0.105	0.0735	0.021	0.189	0.147	0.0105	0.021	0.0525	0.0105	33	41
146	0.1275	0.204	0.2295	0.1785	0.1275	0.1275	0.306	0.0765	0.1275	0.2805	0.2295	0.306	0.102	0.1275	23	24
147	0.1701	0.0486	0.3159	0.1215	0.0972	0.1458	0.0486	0.1944	0.2916	0.243	0.3159	0.0486	0.3159	0.0486	17	18
148	0	0.105	0.0735	0.063	0.084	0.0735	0.084	0.105	0.084	0.0525	0.1155	0.1155	0.0105	0.105	25	26
149	0.0945	0.1575	0.084	0	0	0.021	0.0735	0.105	0.252	0.147	0	0	0.105	0.0105	54	50
150	0.0945	0.1575	0.0735	0.042	0.063	0.0105	0.0735	0.105	0.147	0.189	0.0105	0.021	0.0525	0.0105	52	45
151	0.0945	0.1575	0.21	0.042	0.084	0.0105	0	0.105	0.021	0.189	0.0105	0	0.1155	0.0105	44	39
152	0.0945	0.1575	0.084	0	0.084	0.021	0.0735	0.105	0.189	0.147	0.0105	0.021	0.0525	0.0105	48	46
153	0	0.1575	0.0735	0.042	0.084	0.021	0.0735	0.105	0.252	0.147	0.0105	0.021	0.0525	0.0105	57	50
154	0.1323	0.2205	0.294	0.0588	0	0.0147	0.1911	0.0294	0.3528	0.0882	0.0441	0.0294	0	0.0147	41	50
155	0.0945	0.1575	0.084	0.042	0.084	0.021	0.0735	0.2625	0	0.147	0.0105	0.021	0.0525	0	38	40
156	0	0.1575	0.084	0.042	0.084	0.021	0.126	0.105	0.189	0.147	0.0105	0.021	0.0525	0.0105	41	47
157	0.0945	0.0525	0.105	0	0.084	0.021	0.0735	0.105	0.189	0.147	0.0105	0.105	0.0525	0.0105	31	35
158	0.0126	0.0126	0.0054	0.0108	0.009	0.0126	0.0144	0.018	0.0144	0.009	0.027	0.0198	0.0018	0.018	14	14
159	0.021	0.1575	0.0735	0	0.168	0.021	0.0735	0.105	0.189	0.147	0.0105	0.021	0.0525	0.0105	45	48

No.	Composition[b] (mmol g-support^{-1})														Observed C_4H_6 yield[c] (%)	Predicted C_4H_6 yield[d] (%)
	Mg	Zn	Cu	Ag	Ni	Al	La	Y	Hf	Zr	Cr	Ga	Nb	Mo		
160	0.0945	0.1575	0.084	0	0.105	0.021	0.0735	0.084	0.189	0.147	0.0105	0.021	0.0525	0.0105	44	46
161	0.0945	0.1575	0.084	0.0105	0.084	0.021	0.0735	0.105	0.189	0.147	0	0.021	0.0525	0.0105	42	47
162	0.0498	0.0747	0.3486	0.2988	0.0747	0.1494	0.0996	0.2241	0.1494	0.1743	0.0747	0.1743	0.3486	0.249	21	16
163	0.168	0.036	0.096	0.036	0.192	0.012	0.084	0.132	0	0.156	0.132	0.06	0.072	0.024	24	23
164	0	0.1575	0.0735	0.042	0.084	0.021	0.0735	0.105	0.252	0.147	0.0105	0.021	0.0525	0.0105	50	50
165	0	0.1575	0.084	0.042	0.084	0.021	0.126	0.105	0.252	0.084	0.0105	0.021	0.0525	0.0105	51	49
166	0	0.1575	0.0735	0.042	0.084	0.021	0.0735	0.105	0.252	0.147	0.0105	0.021	0.0525	0.0105	52	50
167	0.1323	0.147	0.1029	0	0.1176	0	0.1911	0.0294	0.3528	0.2058	0.0441	0.1176	0.0147	0.0147	51	46
168	0.078	0.1092	0.078	0.1092	0.1248	0.1404	0.1248	0.078	0.1092	0.0468	0.1872	0.0468	0.1404	0.1872	24	21
169	0.1323	0.2205	0.1029	0	0.0588	0.0294	0.1029	0.0294	0.3528	0.2058	0.0441	0.0294	0.147	0.0147	44	48
170	0.0945	0.1575	0.0735	0	0	0.021	0.0735	0.147	0.252	0.147	0	0.021	0.0525	0.0105	36	51
171	0.0945	0.1575	0.084	0.042	0.084	0.021	0	0.105	0.252	0.147	0.0105	0.021	0.021	0.0105	48	49
172	0.0945	0.126	0.0735	0.042	0.084	0.021	0	0.2625	0.252	0	0.0105	0.021	0.0525	0.0105	49	41
173	0	0.1575	0.084	0.063	0.084	0.021	0.0735	0.105	0.0735	0.0525	0.1995	0.1155	0.0105	0.0105	38	33
174	0	0.1575	0.084	0.042	0.084	0.021	0.126	0.105	0.189	0.147	0.0105	0.021	0.0525	0.0105	48	47
175	0.0945	0.1575	0.084	0	0.084	0.021	0.0735	0.105	0.189	0.147	0.0105	0.021	0.0525	0.0105	53	46
176	0	0.1575	0.0735	0.042	0.084	0.021	0.0735	0.105	0.252	0.147	0.0105	0.021	0.0525	0.0105	50	50
177	0	0.1575	0.0735	0.105	0.084	0.021	0.0735	0.042	0.252	0.147	0.0105	0.021	0.0525	0.0105	50	50

[a] The data were curated from a previous study[5]; [b] Catalysts comprise up to 14 elements co-supported on SBA-15 with varied compositions; [c] Measured at 400 °C and 21.8 mL min^{-1} of 8.4% ethanol in Ar; [d] Obtained using a Huber regressor with eight selected first-order features.

Table S4: Dataset for three-way catalysis[a]

No.	Nanoparticle[b]	Observed T_{50}[c] (°C)	Predicted T_{50}[d] (°C)
1	RhPd	252	262
2	RhPt	266	290
3	CuRh	279	279
4	RuPd	309	311
5	CuPd	319	317
6	CuRhPd	272	269
7	RuRhPd	277	275
8	NiRhPd	279	279
9	FeRhPd	283	268
10	CuRhPt	287	288
11	CoRhPd	290	267
12	NiRhPt	291	296
13	CoRhPt	291	289
14	FeRhPt	294	289
15	RuRhPt	295	295
16	RhPdPt	296	278
17	FeRuPd	309	328
18	CoRuPd	324	329
19	CoPdPt	332	332
20	CuPdPt	333	329
21	NiRuPd	341	341
22	FePdPt	366	330
23	CoRuPt	367	350
24	FeRhPdPt	262	279
25	CrCuRhPd	267	278
26	CuRhPdPt	269	279
27	FeCoRhPd	271	271
28	FeCuRhPd	272	272
29	CoCuRhPd	273	273
30	MnFeRuRh	290	290
31	CrMnPdIr	338	337
32	CrNiPdAg	367	350
33	CoRhPdAgPt	266	289
34	CoNiRuRhPd	272	286

No.	Nanoparticle[b]	Observed T_{50}[c] (°C)	Predicted T_{50}[d] (°C)
35	CoNiRhPdPt	274	285
36	RhPdAgIrPt	276	291
37	FeCoRuRhIr	293	294
38	FeCoNiPdPt	293	339
39	CrMnCuRhAg	296	296
40	NiRhPdInPt	296	296
41	CrRuRhPdPt	298	287
42	FeNiRhPdPt	301	287
43	MnRuRhPdPt	307	286
44	RuRhPdAgPt	309	291
45	CrMnCuPdPt	321	337
46	FeCoRhPdPt	326	282
47	MnCuRuPdPt	330	337
48	CoNiRuAgPt	354	357
49	NiCuRhInIr	355	304
50	CrNiRuPdAg	362	352
51	FeNiAgIrPt	375	357

[a] The data were curated from a previous study[6]; [b] Bimetallic to pentametallic nanoparticles with equimolar compositions are supported on γ-Al_2O_3 at 0.3 wt%; [c] Temperature at 50% NO conversion under stoichiometric conditions; [d] Obtained using a Huber regressor with eight selected first-order features.

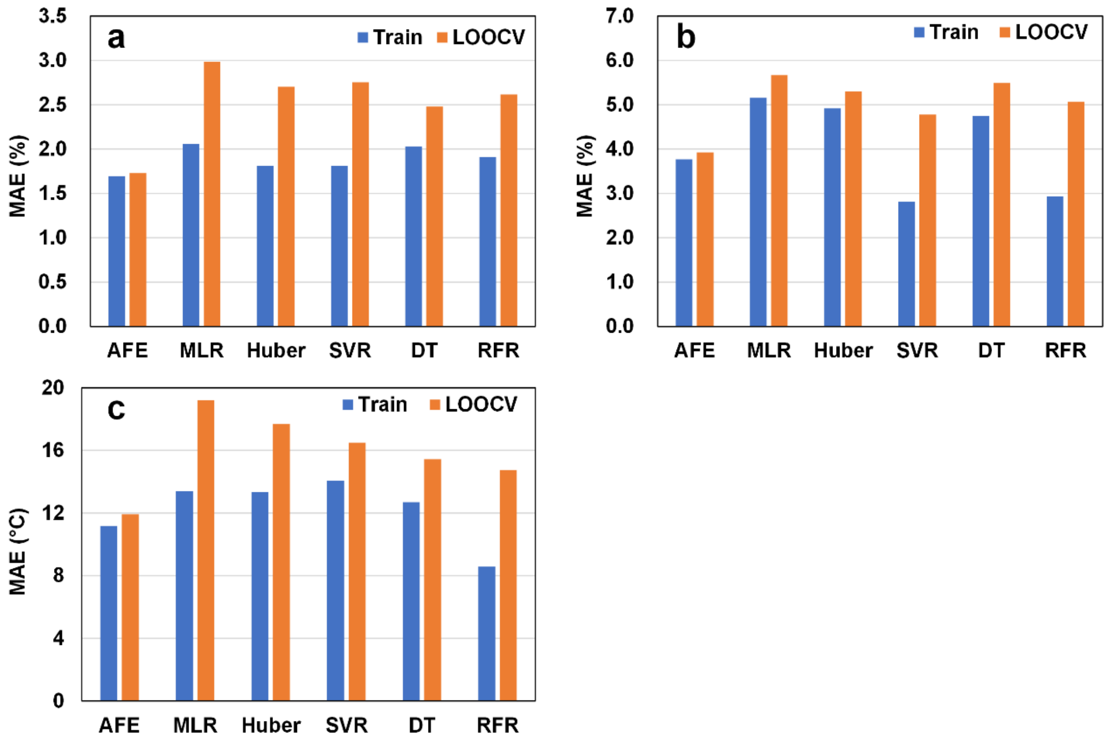

Figure S1: Performance comparison between automatic feature engineering (AFE) and typical regression methods. Mean absolute error (MAE) values in training and leave-one-out cross-validation (LOOCV) are compared across AFE, multiple linear regression (MLE), Huber regression (Huber), support vector regression (SVR), regression tree (DT), and random forest regression (RFR) for predicting (**a**) C_2 yields in the oxidative coupling of methane (OCM), (**b**) butadiene yields in ethanol conversion, and (**c**) light-off temperatures for NO conversion in three-way catalysis. In AFE, eight features that minimized the MAE in LOOCV with Huber regression were selected (see Figures 1b–d). In the other methods, catalyst elemental compositions were directly used as explanatory variables, with hyperparameters (if applicable) optimized through a Bayesian search.

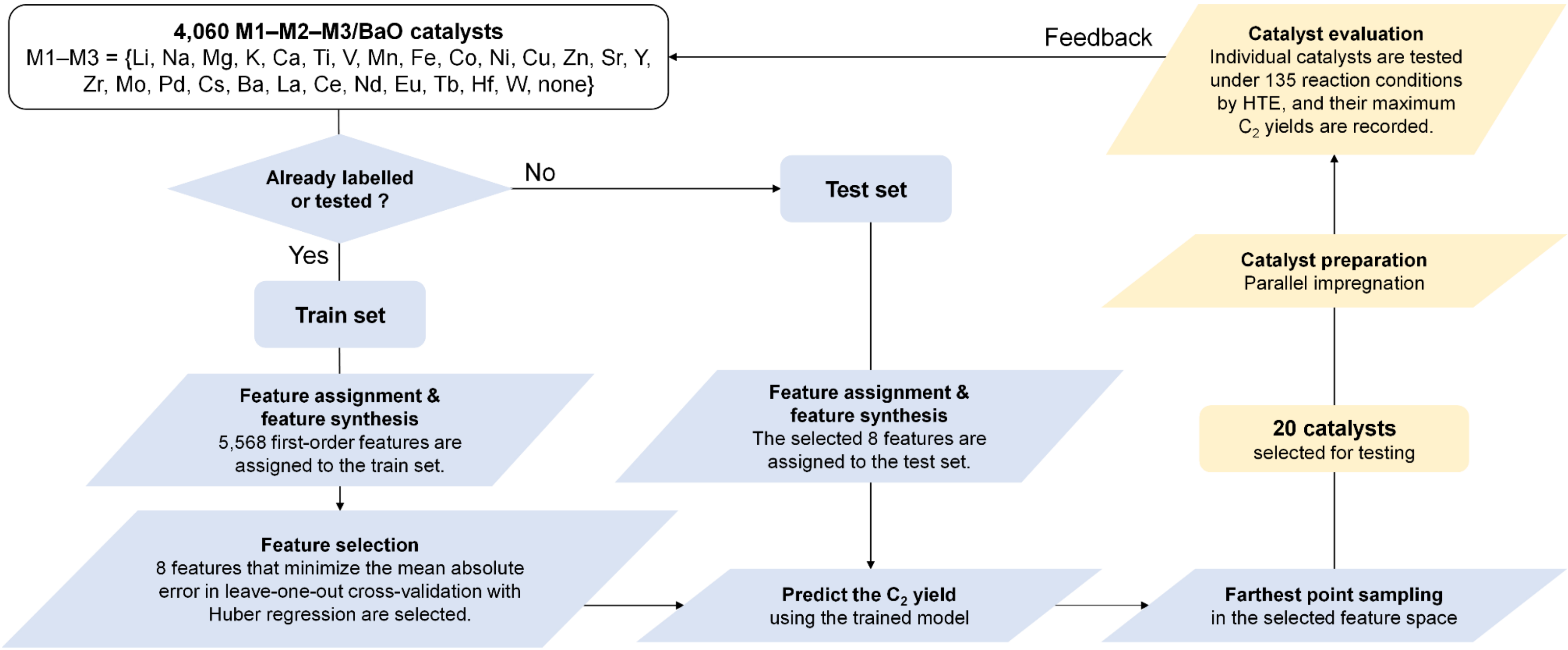

Figure S2: Flowchart of the active learning used for identifying a globally fit model in OCM.

Table S5: Active learning results for the oxidative coupling of methane[a]

No.[b]	M1–M2–M3	Observed C_2 yield (%)	Predicted C_2 yield (%)
1–1	Cs–Cs–Cs	20.6	>30
1–2	Ba–Ba–Ba	5.5	>30
1–3	Fe–Ni–Pd	3.9	–3.7
1–4	Pd–Pd–Pd	7.1	6.6
1–5	Co–Cu–Pd	6.9	–4.7
1–6	Li–Ni–Pd	19.3	–11.4
1–7	K–K–K	9.7	11.0
1–8	Co–Co–Co	0.0	14.7
1–9	Y–Zr–Pd	14.1	2.0
1–10	Mn–Mn–Mn	21.1	12.7
1–11	Nd–Nd–Nd	17.3	–6.2
1–12	K–Cs–Cs	18.1	24.0
1–13	Fe–Ni–Mo	19.7	13.2
1–14	Mo–Mo–Mo	7.0	22.8
1–15	Fe–Pd–W	11.5	0.4
1–16	Cu–Mo–Pd	6.4	–5.8
1–17	Ni–Ni–Ni	5.2	10.4
1–18	Mn–Y–Pd	18.6	–3.1
1–19	K–Mo–La	18.8	17.5
1–20	Sr–Zr–La	20.1	10.8
2–1	W–W–W	21.1	<–30
2–2	Mn–Fe–Pd	13.7	10.0
2–3	Mn–Fe–Mo	17.0	12.6
2–4	Mn–Mn–Zn	16.8	16.7
2–5	Mn–Co–Hf	13.4	7.6
2–6	Co–Pd–Cs	6.9	8.7
2–7	Ni–Mo–Mo	18.3	7.0
2–8	Mg–Zn–Zn	18.6	16.3
2–9	Mn–Ni–Pd	9.7	3.3
2–10	Ni–Zn–Sr	10.0	7.3
2–11	Mn–Fe–Cs	17.5	15.0
2–12	Mn–Mn–Fe	14.6	13.8
2–13	Mg–Mg	17.0	21.1

No.[b]	M1–M2–M3	Observed C_2 yield (%)	Predicted C_2 yield (%)
2–14	Zn–Zn–Ce	18.0	11.2
2–15	K–Fe–Pd	7.3	13.2
2–16	Mn–Co–Cu	7.8	7.8
2–17	Sr–Sr–Cs	18.2	23.9
2–18	Ca–Co–Ni	10.6	8.2
2–19	Mn–Y–Pd	5.1	7.1
2–20	Li–Ni–Pd	5.5	7.8
3–1	Pd–Cs–Cs	0.0	11.2
3–2	Cu–Pd–Cs	5.5	2.6
3–3	Ni–Cs–Cs	7.6	12.8
3–4	Cu–Zn–Zn	7.3	9.0
3–5	Mn–Cs–Cs	10.6	12.9
3–6	Co–Cs–Cs	13.1	14.5
3–7	Mg–Mg–Mn	17.2	12.7
3–8	Pd–W–W	0.0	13.5
3–9	Ba–Ce–Ce	14.5	10.4
3–10	Cu–W–W	7.4	10.5
3–11	Na–K	17.4	16.0
3–12	Na–Cs–Cs	16.5	21.1
3–13	Mn–Mn–W	16.4	13.7
3–14	K–Zn–Cs	18.5	15.2
3–15	Hf–Hf–Hf	17.3	14.5
3–16	Zn–Mo–Pd	11.7	13.8
3–17	Eu–Eu–Eu	19.4	12.9
3–18	Zr–Cs–Cs	17.2	20.0
3–19	Mo–Mo–Mo	18.9	19.1
3–20	Mg–Pd–Ce	0.0	10.3
4–1	Na–Na–Na	16.1	9.3
4–2	Sr–Ba–Ba	15.2	13.3
4–3	Co–Mo–Pd	9.5	7.9
4–4	Na–Mg–Zn	16.0	11.7
4–5	Na–Ca–Ti	14.8	10.9
4–6	Nd	18.3	13.6
4–7	Na–Mg–Ni	2.0	4.5

No.[b]	M1–M2–M3	Observed C_2 yield (%)	Predicted C_2 yield (%)
4–8	K–Cu–Mo	7.1	11.5
4–9	Na–Mg–Mg	14.8	12.0
4–10	Zn–Nd–W	17.2	17.3
4–11	Mn–Mn–Cu	7.0	9.1
4–12	Ca	14.8	12.1
4–13	Mg–Ca–Cu	5.7	4.9
4–14	Mg–Mg–Nd	17.0[c]	21.9
4–15	Mg–Nd	17.5[c]	21.4
4–16	Mg–Nd–Nd	17.4[c]	21.2
4–17	Mn–Mn	11.3[c]	21.1
4–18	Mn	11.4[c]	20.7
4–19	Ca–W	17.7	17.1
4–20	Pd–W–W	0.0	9.8

[a] Observed and predicted C_2 yields acquired in the course of active learning; [b] 20 catalysts were added per cycle, where Nos. 1–18 are the catalysts selected via farthest point sampling in the feature space and Nos. 19 and 20 are the catalysts which exhibited the highest absolute errors during training in the previous cycle; [c] The farthest point sampling was limited to the catalysts with the predicted C_2 yield above 15%.

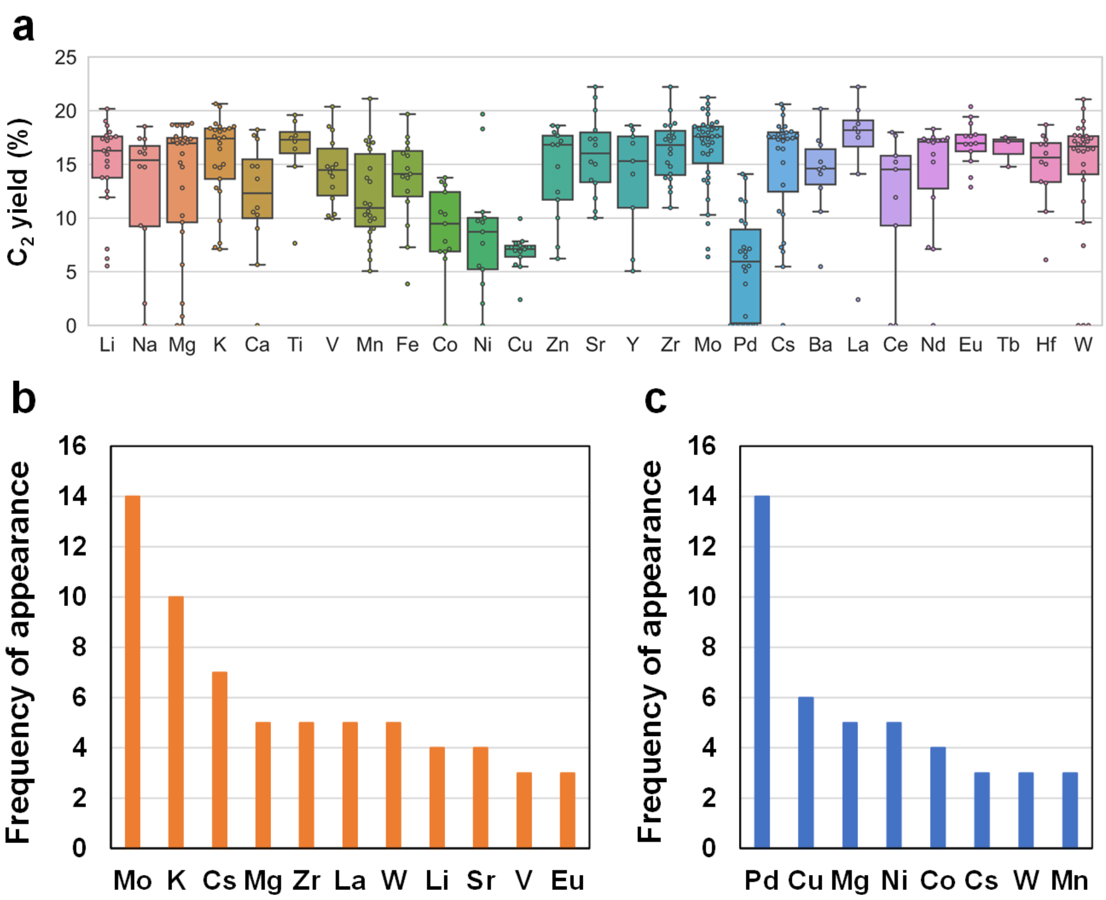

Figure S3: Statistical analysis of catalyst performance with respect to individual elements. (**a**) C_2 yield distribution for each element shown in the box and swarm plots, where the line: the median value, the box: the interquartile range (IQR), and the whisker: 1.5 times the IQR with any data points beyond the whisker regarded as outliers. Elements that are frequently present in the catalysts with a C_2 yield (**b**) above 18% and (**c**) below 7%. Mg appears in both the rankings.

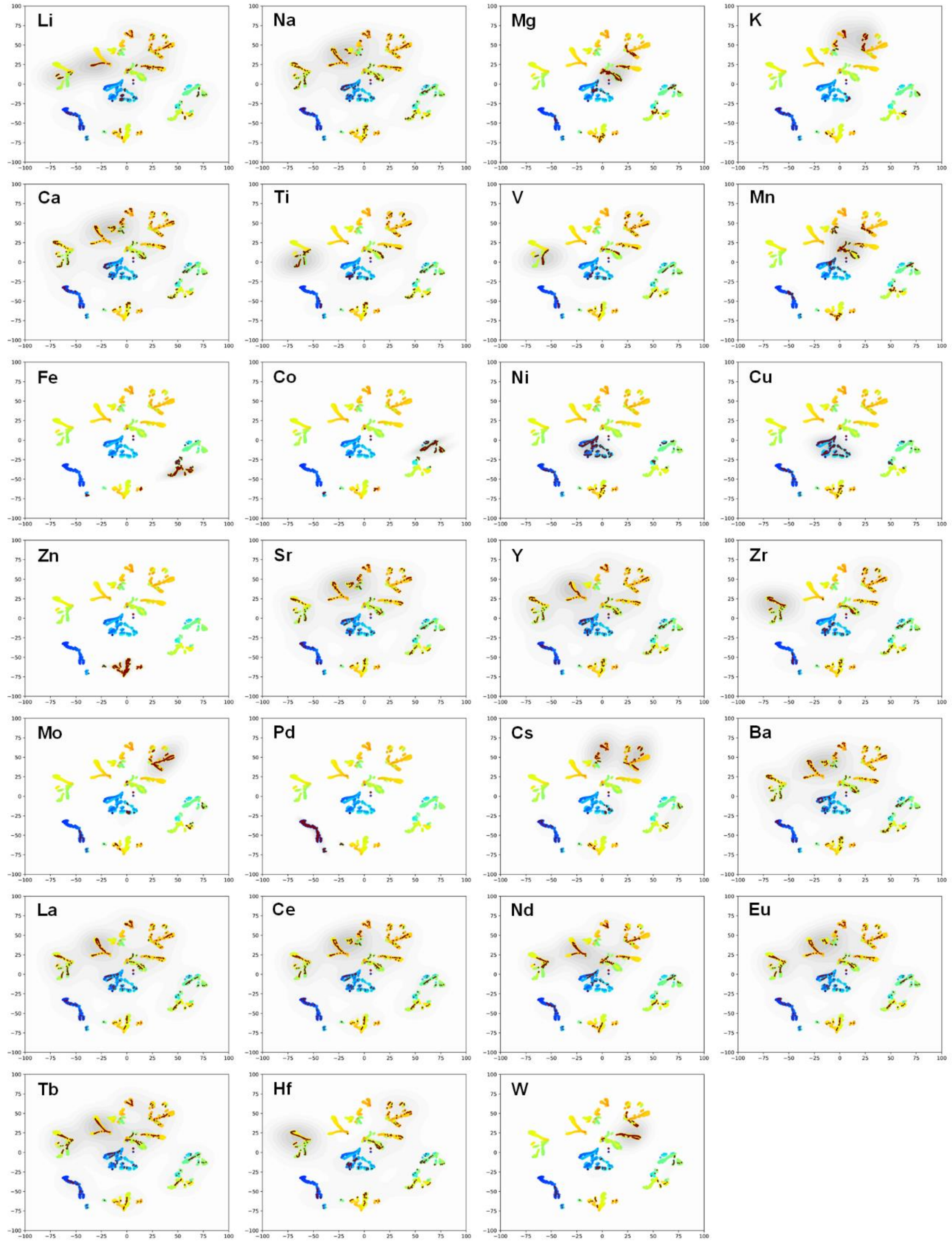

Figure S4: Element-wise distribution in the feature space. The feature space of the latest model is visualized by t-SNE. The black dots represent catalysts containing a specified element with the estimated Gaussian kernel density. The color corresponds to the predicted C_2 yield in the range of 25% (red) to 0% (blue).

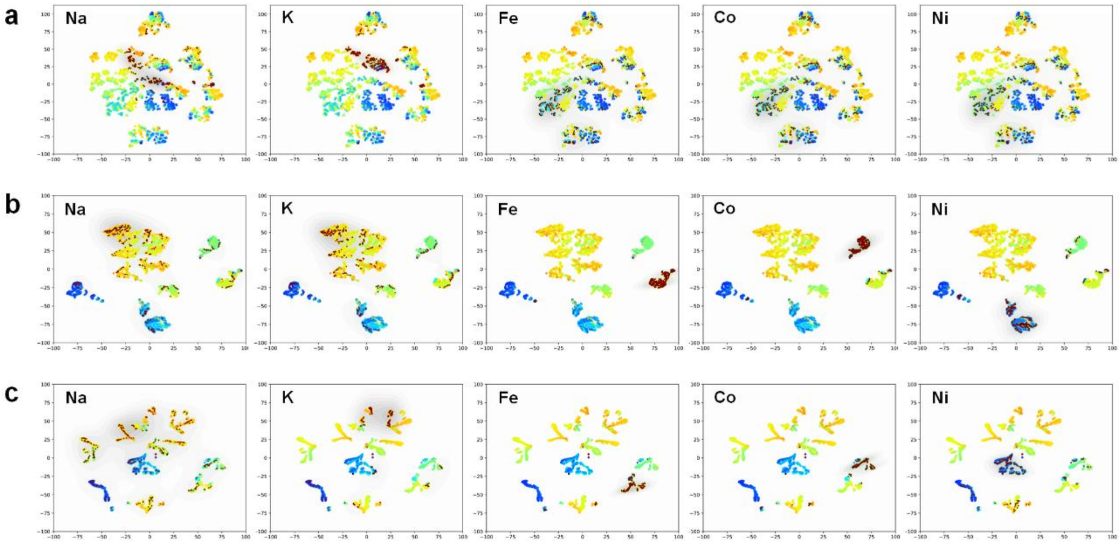

Figure S5: Development of machine perception. In **a–c**, all the primary features, the eight selected first-order features for the initial training dataset, and the eight selected first-order features at the end of the active learning are used as the input of t-SNE visualization, respectively. The distribution of selected elements in the respective t-SNE spaces is presented with black dots and Gaussian kernel density estimation.

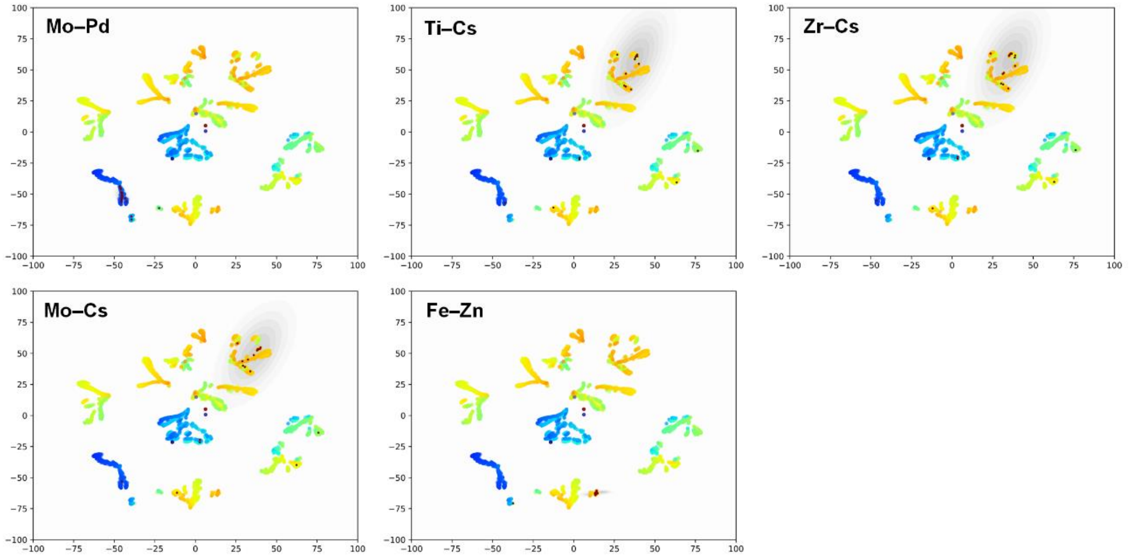

Figure S6: Visualization of combinatorial rules using t-SNE. The black dots represent catalysts containing a specified binary combination with the estimated Gaussian kernel density. The color corresponds to the predicted C_2 yield in the range of 25% (red) to 0% (blue).

Table S6: Performance of selected catalysts in the oxidative coupling of methane[a]

M1–M2–M3	Observed C_2 yield (%)
Fe–Fe–Fe	20.4
K–Fe–Zn	17.8
Fe–Zn	18.9
Fe	19.4
K–Co–Zn	10.4
Li–Fe–Zn	14.0
Cs	15.0
Co–Zn	11.8
Ca–Fe–Zn	19.6
Mg–K	15.0
Ti	17.3
K–Zn	16.5
Na	15.8
Mo	20.1
Ce	18.6
Fe–Ni–Zn	15.3
Eu–Eu	18.1
K	16.8
Zn–Zn–Zn	17.5
Mo–Mo–Ce	13.0
Zn–Mo	19.3
Fe–Mo–Eu	19.8
V–Zn–Zn	12.5
Ti–Mo–Mo	14.1
Ti–Zn–Mo	18.7
V–Hf–Hf	16.1
Fe–Mo–Mo	18.0
Mo–Mo–W	18.9
Zr–La–La	19.6
V–La–La	18.9
Ti–V–W	16.3
Ti–W–W	19.6
Ti–La–La	19.3

M1–M2–M3	Observed C_2 yield (%)
Na–Tb–Tb	16.0
Li–Ti–Zr	16.2
Fe–Mo–La	20.1

[a] 36 catalysts, chosen from a subset of catalysts with predicted C_2 yields $\geq$ 15% using farthest point sampling, were experimentally tested.

Table S7: Model performance in various cases[a]

No.	Feature set[b]	Feature selection[c]	Regression method[d]	R^2_{train}	R^2_{CV}[e]
1	S	n.a.	Huber	0.27	−1.18
2	S	n.a.	RFR	0.72	−0.49
3	S	8 (GA)	Huber	0.47	0.14
4	S	8 (GA)	RFR	0.50	0.18
5	X_0	n.a.	Huber	0.74	−1.41
6	X_0	n.a.	RFR	0.75	−0.22
7	X_0	8 (GA)	Huber	0.52	0.45
8	X_0	8 (GA)	RFR	0.90	0.38
9	X_0	8 (GA)	MLR	0.53	0.38
10	X_0	8 (GA)	SVR	0.36	0.36
11	X_0	8 (SFS)	Huber	0.36	0.28
12	X_0	8 (SFS)	RFR	0.52	0.10
13	X_1	8 (GA)	Huber	0.66	0.58
14	X_1	4 (GA)	Huber	0.60	0.44
15	X_1	12 (GA)	Huber	0.72	0.64
16	X_1	8 (GA)	RFR	0.93	0.43
17	X_2	8 (GA)	Huber	0.73	0.68
18	X_3	8 (GA)	Huber	0.74	0.70

[a] Demonstrated for the OCM dataset of Table S1 (Nos. 1–69) using the C_2 yield as the target variable; [b] Feature set used as explanatory variables, where S: the chemical composition of catalysts and X_i: the ith-order features; [c] Number of selected features and selection method, where n.a.: no selection, GA: genetic algorithm, and SFS: sequential feature selection; [d] Method of regression, where Huber: Huber regression, RFR: random forest regression, MLR: multiple linear regression, and SVR: support vector regression; [e] Average in 50-fold shuffle split cross-validation (90:10).

Supplementary References

1. Nguyen, T. N. et al. Learning Catalyst Design Based on Bias-Free Data Set for Oxidative Coupling of Methane. *ACS Catal.* **11**, 1797–1809 (2021).
2. Nakanowatari, S. et al. Extraction of Catalyst Design Heuristics from Random Catalyst Dataset and their Utilization in Catalyst Development for Oxidative Coupling of Methane. *ChemCatChem* **13**, 3262–3269 (2021).
3. Takahashi, L. et al. Constructing catalyst knowledge networks from catalyst big data in oxidative coupling of methane for designing catalysts. *Chem. Sci.* **12**, 12546–12555 (2021).
4. Takahashi, K. et al. Catalysis Gene Expression Profiling: Sequencing and Designing Catalysts. *J. Phys. Chem. Lett.* **12**, 7335–7341 (2021).
5. Jayakumar, T. P. et al. Exploration of ethanol-to-butadiene catalysts by high-throughput experimentation and machine learning. *Appl. Catal. A Gen.* **666**, 119427 (2023).
6. Son, S. D. et al. High-throughput screening of multimetallic catalysts for three-way catalysis. *Sci. Technol. Adv. Mater. Methods* DOI:10.1080/27660400.2023.2284130 (2023).
